# The Role of P4HA1 in Multiple Cancer Types and its Potential as a Target in Renal Cell Carcinoma

**DOI:** 10.3389/fgene.2022.848456

**Published:** 2022-06-23

**Authors:** Yang Li, Yu-Zheng Ge, Yiguan Qian, Ke Chen, Feng Zhao, Zhiqiang Qin, Liuhua Zhou, Luwei Xu, Zheng Xu, Quanliang Dou, Ruipeng Jia

**Affiliations:** ^1^ Department of Urology, Nanjing First Hospital, Nanjing Medical University, Nanjing, China; ^2^ Southeast University School of Medicine, Nanjing, China

**Keywords:** P4HA1, pan-cancer analysis, renal cell carcinoma, epithelial–mesenchymal transition, oncogene, prognosis

## Abstract

**Background:** Prolyl 4-hydroxylase subunit alpha 1 (P4HA1) provides the majority of the catalytic site of the active P4H enzyme. Emerging evidence has revealed that P4HA1 participates in the initiation and development of several malignant tumors. However, a pan-cancer analysis of P4HA1 has not been performed.

**Methods:** In this study, we carried out an in-depth analysis of the expression patterns and prognostic value of P4HA1 using the datasets of The Cancer Genome Atlas (TCGA) and Kaplan–Meier Plotter. Genomic and epigenetic alterations of P4HA1 and the correlation of P4HA1 with DNA methylation in different cancers were also analyzed across multiple databases. In addition, the purity-adjusted partial Spearman’s correlation test was utilized to evaluate the correlation between P4HA1 expression and immune cell infiltration. We also further explored the biological function and mechanism of P4HA1 in renal cell carcinoma (RCC).

**Results:** We characterized the expression profiles and prognostic values of P4HA1 in multiple cancer types. P4HA1 expression was increased in clear cell renal cell carcinoma (RCC) compared to adjacent normal tissues, and P4HA1 positively correlated with the overall survival (OS) and disease-free survival (DFS) in papillary RCC. In addition, a positive correlation between P4HA1 expression and immune cell infiltration was observed in clear cell RCC. We also identified a strong correlation between P4HA1 expression and immune checkpoint gene expression, microsatellite instability, and tumor mutation burden in chromophobe RCC. Finally, the results of *in vitro* experiments verified that overexpression of P4HA1 promoted the proliferation, migration, invasion, and epithelial–mesenchymal transition of RCC cells.

**Conclusion:** Overall, our study has suggested that P4HA1 might play a significant role in tumorigenesis in RCC and may be a prognostic biomarker and therapeutic target for several malignant tumors, including RCC.

## Background

Cancer is one of the leading causes of morbidity and mortality worldwide, and it has increased the health and economic burden on society (Sung et al., 2021). Although cancer therapeutic strategies such as surgery, chemotherapy, radiotherapy, immunotherapy, and targeted therapy have achieved some clinical success, poor prognosis and high progression and recurrence rates remain challenging (Siegel et al., 2021). Therefore, identifying reliable diagnostic and prognostic biomarkers and exploring the potential molecular mechanisms of cancer are important.

Prolyl 4-hydroxylases (P4Hs) are tetrameric α2β2 α-ketoglutarate (α-KG)-dioxygenases that are crucial for the appropriate folding of collagen polypeptide chains into stable triple-helical molecules (Myllyharju, 2003; Myllyharju and Kivirikko, 2004; Timmins et al., 2017). There are three main isoforms of the P4HA subunit (P4HA1, P4HA2, and P4HA3), which can form A2B2 tetramers with P4HB, yielding P4H1, P4H2, and P4H3 holoenzymes, respectively (Annunen et al., 1997; Kukkola et al., 2003). P4HA1 is the most crucial isoform. It is necessary for the formation of collagen comprising two identical alpha subunits and two beta subunits (Zou et al., 2017; Xiong et al., 2018). P4HA1 is primarily expressed in the testis and placenta and is involved in peptide bond formation and protein scaffolding (Xiong et al., 2018). However, P4HA1 has also recently been revealed to be involved in the carcinogenesis of different types of cancers. For example, P4HA1 is essential for lung adenocarcinoma (LUAD) cell growth and invasion and may be a valid therapeutic target in LUAD (Robinson et al., 2021). Furthermore, hypoxia can promote the metastasis of glioblastoma multiforme (GBM) *via* the L-Arg/P4HA1 axis, and an increased P4HA1 is associated with poor prognosis and advanced histological grade (Zhu et al., 2021). In primary melanoma, P4HA1 promotes cell adhesion, invasion, and viability and serves as a latent prognostic marker and therapeutic target (Eriksson et al., 2020). The levels of P4HA1 and P4HA2 are significantly increased in uveal melanoma patients with metastatic disease and are related to poor prognosis (Kaluz et al., 2021). P4HA1-HIF1α signaling can function as a regulator of oncogenic activities in pancreatic ductal adenocarcinoma (Cao et al., 2019). These findings indicate that P4HA1 may play a role in the initiation and progression of cancer. However, most studies on the biological role and function of P4HA1 are limited to a particular type of cancer, and a comprehensive pan-cancer study on P4HA1 has not been performed.

In this work, we carried out a pan-cancer analysis of P4HA1 using online databases to systematically profile P4HA1 expression, genetic alteration, association with immune infiltration and DNA methylation, and relevant cellular pathways across various cancers. In addition, the ability of P4HA1 expression to predict prognosis in different cancer types was analyzed to investigate its potential therapeutic utility. We also assessed the expression of P4HA1 in renal cell carcinoma (RCC) tissues and confirmed its biological function and latent mechanism *via* a series of *in vitro* experiments. Therefore, our study highlights the essential role of P4HA1 in tumorigenesis and suggests its utility as a potential and promising biomarker and therapeutic target in various tumors, especially RCC.

## Materials and Methods

### Patients and Tissue Collection

Sixteen paired RCC tumor specimens and adjacent nontumor tissues were obtained from the Department of Urology, Nanjing First Hospital, and placed at −80°C immediately after resection. All RCC samples were diagnosed by two pathologists independently. All patients provided informed consent, and this work was approved by the Institutional Research Ethics Committee of Nanjing First Hospital.

### Cell Culture and Transfection

The RCC cell lines 786-O and ACHN were obtained from the American Type Culture Collection (Manassas, VA, United States). 786-O cells were cultured in RPMI-1640 (Thermo Scientific, China), and ACHN cells were cultured in DMEM (Thermo Scientific). All cells were cultured with antibiotics (1% penicillin–streptomycin) and 10% fetal bovine serum (FBS, Invitrogen, Carlsbad, CA, United States). The cells were cultured at 37°C in a humidified atmosphere of 5% CO_2_. The P4HA1 overexpression plasmid (OE-P4HA1) and corresponding negative control were designed and purchased from GenePharma (GenePharma Co., China). Transfection was performed using Lipofectamine 3000 (Invitrogen) according to the manufacturer’s protocol.

### RNA Extraction and Quantitative Real-Time PCR

Total RNA was extracted from tissues or cell lines utilizing TRIzol reagent (Invitrogen) according to the manufacturer’s protocol. The quality and quantity of RNA were assessed by Nanodrop 2000 spectrophotometry (Thermo Scientific). The total RNA was reverse-transcribed to complementary DNA using the PrimeScript RT reagent (Takara, Japan). We used SYBR Premix Ex Taq II (Takara) to detect the relative expression of P4HA1 and other genes using a LightCycler 480 real-time PCR instrument (Roche). The relative expression level was calculated by the 2^−ΔΔCt^ method. *GADPH* was used for standardization. The detailed primer sequences are shown in Table 1.

**TABLE 1:** Sequences of primers for qRT-PCR.

Name		Sequence
P4HA1	Forward	5′- AGT​ACA​GCG​ACA​AAA​GAT​CCA​G -3′
	Reverse	5′- CTC​CAA​CTC​ACT​CCA​CTC​AGT​A -3′
E-cadherin	Forward	5′- GTA​CTT​GTA​ATG​ACA​CAT​CTC -3′
	Reverse	5′- TGC​CAG​TTT​CTG​CAT​CTT​GC -3′
N-cadherin	Forward	5′- CGA​ATG​GAT​GAA​AGA​CCC​ATC​C -3′
	Reverse	5′- GGA​GCC​ACT​GCC​TTC​ATA​GTC​AA -3′
Vimentin	Forward	5′- GAA​GAG​AAC​TTT​GCC​GTT​GAA​G -3′
	Reverse	5′- ACG​AAG​GTG​ACG​AGC​CAT​T -3′
GAPDH	Forward	5′- GCT​TCG​GCA​GCA​CAT​ATA​CTA​AAA​T-3′
	Reverse	5′- CGC​TTC​ACG​AAT​TTG​CGT​GTC​AT -3′

### Western Blotting

Total protein from the tissues and cell lines was extracted using RIPA lysis buffer (Beyotime, China). Equal amounts of total protein were separated by 10% SDS-PAGE and transferred onto PVDF membranes (Millipore, United States). Then, the membranes were blocked with 5% nonfat milk and incubated with primary antibodies against P4HA1 (1:1000, ab244400, Abcam, United States), E-cadherin (1:1000, ab231303, Abcam), N-cadherin (1:1000, ab98952, Abcam), vimentin (1:1000, ab92547, Abcam), and GAPDH (1:20000, 10494-1-AP, Proteintech, United States) overnight at 4°C. After incubation with an HRP-conjugated secondary antibody (1:5000, Proteintech) for 2 h, the protein bands were exposed using a molecular imager system (Bio-Rad Laboratories).

### EdU Assay

Cell proliferation was assessed using the Cell-LightTM EdU Apollo^®^ 567 (RiboBio, China). The transfected cells were plated into 96-well plates at 3 × 10^4^ cells per well. After 24 h, the cells were treated with 100 mM of EdU reagent for 2 h at 37°C. Then, the cells were fixed with 4% paraformaldehyde (Sigma, United States) for 20 min. Five fields were randomly selected under a fluorescence microscope (Olympus, Japan).

### Transwell Migration and Invasion Assay

Transwell chambers (Millipore) with 8-μm-pore polycarbonate filters were used to investigate the effect of P4HA1 on cell migration and invasion. Approximately 1 × 10^5^ cells in 100 μl serum-free medium were placed in the upper chamber that was (invasion) or was not (migration) precoated with Matrigel (Becton, United States), and 600 μl culture medium containing 10% FBS was placed into the lower chamber. After 36 h at 37°C, Matrigel and cells were removed from the upper surface of the filters. Then, the cells on the bottom of the filters were fixed with 4% paraformaldehyde and stained with 0.5% crystal violet (Beyotime). The cells were counted in five randomly selected fields under an inverted microscope (Olympus IX90) at ×200 magnification.

### Immunofluorescence Assay

After transfection, 786-O and ACHN cells were fixed with 4% paraformaldehyde, permeabilized in 0.3% Triton X-100, and blocked in 5% serum. Then, the cells were incubated overnight at 4°C with E-cadherin (1:1000, ab231303, MA, United States), N-cadherin (1:500, ab98952, MA, United States), or vimentin (1:500, ab92547, MA, United States) antibodies. Then, a secondary fluorescent antibody was added, and the cells were incubated at room temperature for another 2 h. The cells were stained with DAPI staining solution (Beyotime) for 10 min. The images were photographed using a confocal microscope (Leica, United States) at ×400 magnification.

### Analysis of Prolyl 4-Hydroxylase Subunit Alpha 1 Expression in Cancers

We used the “Gene_DE” module of Tumor Immune Estimation Resource, version 2 (TIMER2, http://timer.cistrome.org/) (Li et al., 2017), the “Expression Analysis-Box Plots” module of Gene Expression Profiling Interactive Analysis, version 2 (GEPIA2, http://gepia2.cancer-pku.cn/) (Tang et al., 2017), and Genotype-Tissue Expression (GTEx, http://gtexportal.org) to assess the differential expression of P4HA1 between tumor and normal tissues. Moreover, we used the “Pathological Stage Plot” module of GEPIA2 to create violin plots of P4HA1 expression in distinct pathological stages across The Cancer Genome Atlas (TCGA) datasets. We chose log2 [TPM+1]-transformed expression data for plotting. The UALCAN (http://ualcan.path.uab.edu/analysis-prot.html) portal was used to analyze protein expression levels in the Clinical Proteomic Tumor Analysis Consortium (CPTAC) module (Chandrashekar et al., 2017) among distinct tumor tissues and adjacent normal tissues.

### Survival Analysis

We performed overall survival (OS) and disease-free survival (DFS) analyses based on P4HA1 expression in different tumor types in TCGA datasets using the “Survival Map” module of GEPIA2 (Tang et al., 2019). We divided the patients into high-expression and low-expression cohorts based on the median expression. The hazard ratio, 95% confidence interval, and log-rank *p* values were also calculated.

### Genomic Alterations of Prolyl 4-Hydroxylase Subunit Alpha 1 in Cancer

The alteration frequency, mutation type, and copy number alteration of P4HA1 in different tumors were analyzed using the “Cancer Types Summary” module of cBioPortal (https://www.cbioportal.org/) (Cerami et al., 2012; Gao et al., 2013). We also used the “Mutation” module of cBioPortal to acquire the three-dimensional (3D) structure of the P4HA1 protein. Moreover, the “Comparison” module of cBioPortal was applied to generate the Kaplan–Meier plots with log-rank *p* values to analyze OS, DFS, progression-free survival (PFS), and disease-specific survival (DSS) based on P4HA1 genetic alteration.

### Correlation Between Prolyl 4-Hydroxylase Subunit Alpha 1 and Immune Cell Infiltration

We used the “Immune-Gene” module of TIMER2 to evaluate the correlation between P4HA1 expression and immune cell infiltration. B cells, CD4^+^ T cells, CD8^+^ T cells, neutrophils, macrophages, dendritic cells, and cancer-associated fibroblasts (CAFs) were assessed. The EPIC, MCPCOUNTER, XCELL, TIDE, CIBERSORT, CIBERSORT-ABS, and QUANTISEQ algorithms were applied for estimations. Spearman’s correlation test is applicable to data with two columns of variables and hierarchical linear relationship. Both P4HA1 expression level and immune cell infiltration are variables; therefore, the purity-adjusted partial Spearman’s correlation test was utilized to evaluate the correlation between P4HA1 expression and immune cell infiltration.

### Enrichment Analysis of Prolyl 4-Hydroxylase Subunit Alpha 1-Related Genes

The STRING (https://string-db.org/) tool was used to predict the protein–protein interaction networks (Szklarczyk et al., 2019). The main parameters were as follows: “low confidence (0.150),” the meaning of network edges to “evidence,” and the maximum number of interactors to show “no more than 50 interactors.” The accuracy of the list was consistent with text mining and experimental evidence.

We used the “Similar Gene Detection” module of GEPIA2 to identify the top 100 P4HA1-related genes in TCGA datasets. We also utilized the “correlation analysis” module of GEPIA2 to calculate the correlation between P4HA1 and related genes. The correlation coefficient and *p* value are displayed in the corresponding figure panels.

The “Gene_Corr” module of TIMER2 was applied to display the heatmaps of expression of P4HA1-related genes in different tumor types. The *p* value and partial correlation are displayed in the corresponding figure panels. Intersection analysis to evaluate P4HA1 binding proteins and interacting genes was performed using the Venn diagram viewer (http://bioinformatics.psb.ugent.be/webtools/Venn/).

We combined the two sets of data to conduct Kyoto Encyclopedia of Genes and Genomes (KEGG) pathway analysis and gene ontology (GO) enrichment analysis. The enriched pathways were visualized with the “ggplot2” and “clusterProfiler” R packages (R-4.0.4, 64-bit, https://www.r-project.org/). The data for biological processes, cellular components, and molecular functions in the GO enrichment analysis were visualized with the “cnetplots” R package. A two-tailed *p* < 0.05 was regarded as statistically significant.

### Statistical Analysis of *In Vitro* Experiments

We conducted statistical analysis using GraphPad Prism 8.0 (GraphPad, United States). Student’s *t*-tests were utilized to analyze the statistical significance. The data were displayed as the mean ± SD. A *p* < 0.05 was considered statistically significant. For each group of experiments, we had conducted three independent repeated assays. Only when at least three repeated experiments presented the same conclusion after conducting statistical analysis, we regarded the experimental results as persuasive. In this article, we chose one experimental result for drawing the display.

## Results

### Analysis of Prolyl 4-Hydroxylase Subunit Alpha 1 Expression in Cancer

We carried out a comprehensive analysis toward the latent biological role of P4HA1 (NM_001017962.3 for mRNA or NP_001017962.1 for protein, Supplementary Figure S1A) across tumor types. First, we found that the P4HA1 protein structure was conserved among species and universally consisted of the P4Ha_N (pfam08336) domain and the 2OG-FeII_Oxy_3 (cll7304) domain (Supplementary Figure S1B). We also analyzed the evolutionary correlation of P4HA1 in distinct species. The phylogenetic tree data are displayed in Supplementary Figure S2. These results indicated that P4HA1 might play a vital role in various biological processes.

We, next, evaluated the relative expression of P4HA1 in normal tissues and cell lines. Based on the Human Protein Atlas (HPA), GTEx, and Function Annotation of the Mammalian Genome-5 datasets, P4HA1 was most highly expressed in the vagina, followed by the skeletal muscle and breast (Supplementary Figure S3A). We saw an enhanced tissue expression pattern in the vagina. We further found that P4HA1 was ubiquitously expressed in immune cells, and it had low immune cell specificity (Supplementary Figure S3B). Furthermore, we used the HPA/Monaco/Schmiedel datasets to evaluate the P4HA1 expression in distinct blood cells. *P4HA1* also exerted a low RNA blood cell type specificity (Supplementary Figure S3C).

**FIGURE 1:**
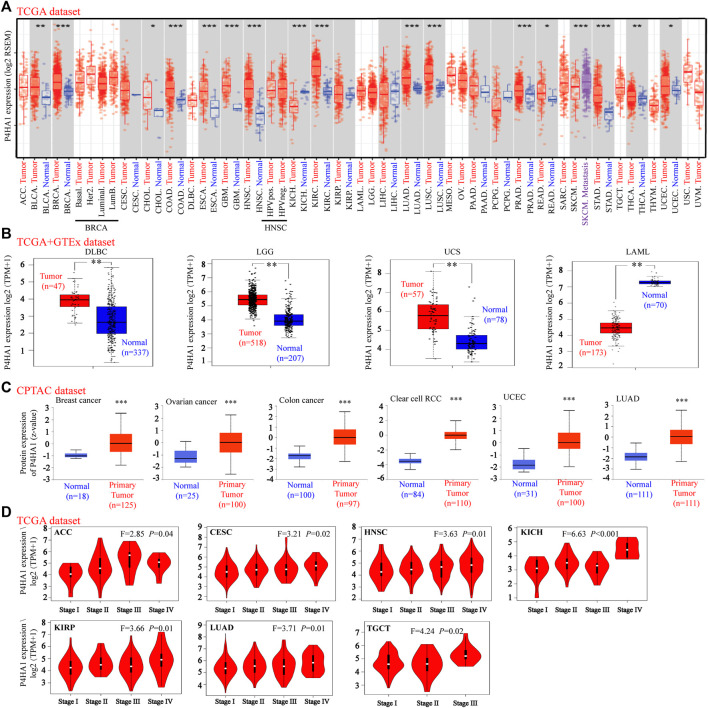
Expression level of P4HA1 in distinct cancers and pathological stages. **(A)** Expression level of the P4HA1 in distinct tumors or specific tumor subtypes was analyzed by TIMER2.0 (http://timer.cistrome.org/). **(B)** We analyzed the expression level of P4HA1 in DLBC, LGG UCS, and LAML in the TCGA project and GTEx database. **(C)** Total protein expression level of P4HA1 in BRCA, OV, colon cancer, ccRCC, UCEC, and LUAD was analyzed according to the CPTAC dataset (http://ualcan.path.uab.edu/). **(D)** On the basis of the TCGA dataset, the expression level of P4HA1 was analyzed by the main pathological stages (stage I, stage II, stage III, and stage IV) of ACC, CESC, HNSC, KICH, KIRP, LUAD, and TGCT. *p < 0.05, **p < 0.01, and ***p < 0.001; log2 (TPM + 1) was applied for log-scale.

TIMER 2.0 was used to calculate the expression pattern of P4HA1 in different tumor types. As shown in Figure 1A, the levels of P4HA1 were higher in bladder cancer (BLCA), BRCA, cholangiocarcinoma (CHOL), colon adenocarcinoma (COAD), esophageal carcinoma (ESCA), GBM, head and neck squamous cell carcinoma (HNSC), clear cell RCC, LUAD, lung squamous cell carcinoma (LUSC), prostate adenocarcinoma (PRAD), rectal adenocarcinoma (READ), stomach adenocarcinoma (STAD), thyroid carcinoma (THCA), and UCEC than in the adjacent normal tissues. However, the expression of P4HA1 was decreased in the chromophobe RCC tissues compared to the normal tissues.

Because several tumor types did not have matching normal tissue data in TCGA, GEPIA was used to further analyze the expression of P4HA1 between tumor and normal tissues. P4HA1 expression was increased in diffuse large B-cell lymphoma (DLBCL), brain lower grade glioma (LGG), and uterine carcinosarcoma (UCS) tumor tissues compared to the adjacent normal tissues, whereas it was decreased in acute myeloid leukemia (AML) (Figure 1B). In addition, there was no significant difference in the P4HA1 expression between tumor and normal tissues in patients with adrenocortical carcinoma (ACC), OV, pheochromocytoma and paraganglioma (PCPG), sarcoma (SARC), testicular germ cell tumor (TGCT), or thymoma (THYM) (Supplementary Figure S4A).

We also used the CPTAC database to evaluate the total protein level of P4HA1 in breast cancer, OV, colon cancer, clear cell RCC, UCEC, and LUAD. The total protein levels of P4HA1 in these six tumor types were much higher than those in the negative controls (Figure 1C). The Oncomine dataset was used to perform a pooling analysis of distinct studies. The results demonstrated that P4HA1 was upregulated in brain and CNS cancer, breast cancer, CRC, head and neck cancer, kidney cancer, lung cancer, pancreatic cancer, and SARC compared with the normal tissues (Supplementary Figure S5).

Moreover, the “Pathological Stage Plot” module of GEPIA2 was used to evaluate the relationship between P4HA1 expression and the pathological stages of tumors. We discovered that the expression of P4HA1 was related to stage in several cancers, including ACC, CESC, HNSC, chromophobe RCC, papillary RCC, LUAD, and TGCT (Figure 1D) but not in other cancers (Supplementary Figures S5B–E).

Moreover, the corresponding methods could be found in Supplementary Materials.

### Survival Analysis of Prolyl 4-Hydroxylase Subunit Alpha 1

**FIGURE 2:**
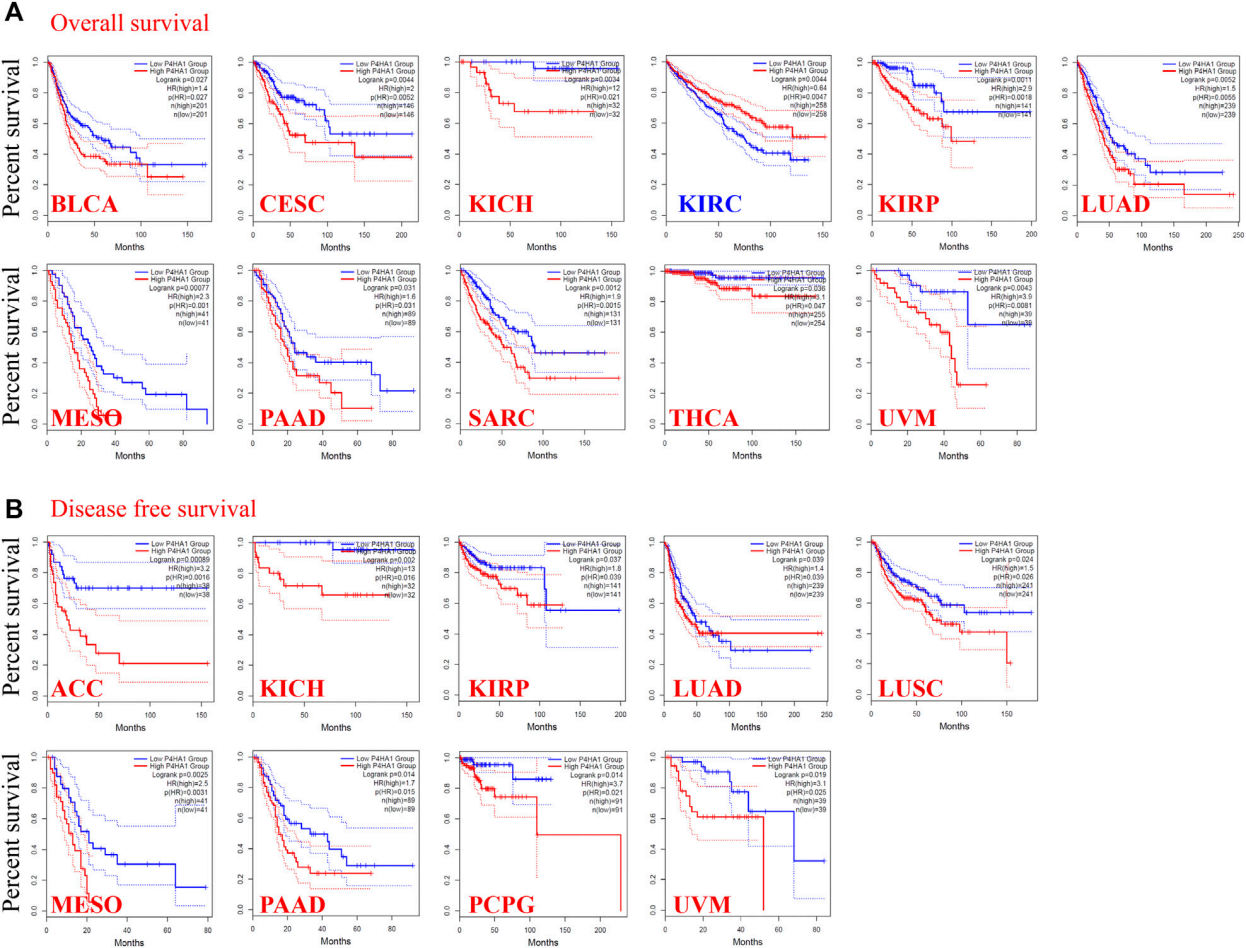
Correlation between the P4HA1 expression level and survival prognosis of different tumors in the TCGA dataset. **(A,B)** We used the GEPIA2 tool (http://gepia.cancer-pku.cn/) to conduct overall survival (OS) **(A)** and disease-free survival (DFS) **(B)** analyses of different tumors in TCGA by P4HA1 expression level.

To determine the prognostic value of P4HA1 expression across cancer types, we divided the tumors into high-expression and low-expression cohorts based on the median expression of P4HA1 and analyzed its relationship with survival in the TCGA and GEO databases. High expression of P4HA1 was related to poor OS in BLCA, CESC, chromophobe RCC, papillary RCC, LUAD, MESO, PAAD, SARC, THCA, and UVM in TCGA (Figure 2A). DFS analysis indicated a correlation between the high expression of P4HA1 and poor prognosis in ACC, chromophobe RCC, papillary RC, LUAD, LUSC, MESO, PAAD, PCPG, and UVM in TCGA (Figure 2B). In addition, low expression of P4HA1 was related to poor OS in clear cell RCC (Figure 2A), which was inconsistent with the mRNA and protein expression patterns.

We used the Kaplan–Meier Plotter to further evaluate the association between P4HA1 and survival. We discovered positive correlations between the high P4HA1 expression and poor OS, distant metastasis-free survival, and relapse-free survival in breast cancer (Supplementary Figure S6A), poor OS and PFS in ovarian cancer (Supplementary Figure S6B), and poor OS in lung cancer (Supplementary Figure S6C). Low expression of P4HA1 was related to poor OS, first progression, and post-progression survival in gastric cancer (Supplementary Figure S6D) and poor PFS in liver cancer (Supplementary Figure S6E). We conducted a meta-analysis to further verify the correlation between P4HA1 expression and prognosis in breast, ovarian, lung, gastric, and liver cancers (Supplementary Figure S7). In addition, a series of subgroup analyses utilizing the selected clinical factors was performed to identify different conclusions (Supplementary Tables S1–S5).

Moreover, the corresponding methods could be found in Supplementary Material.

### Genetic Alteration Analysis of Prolyl 4-Hydroxylase Subunit Alpha 1

**FIGURE 3:**
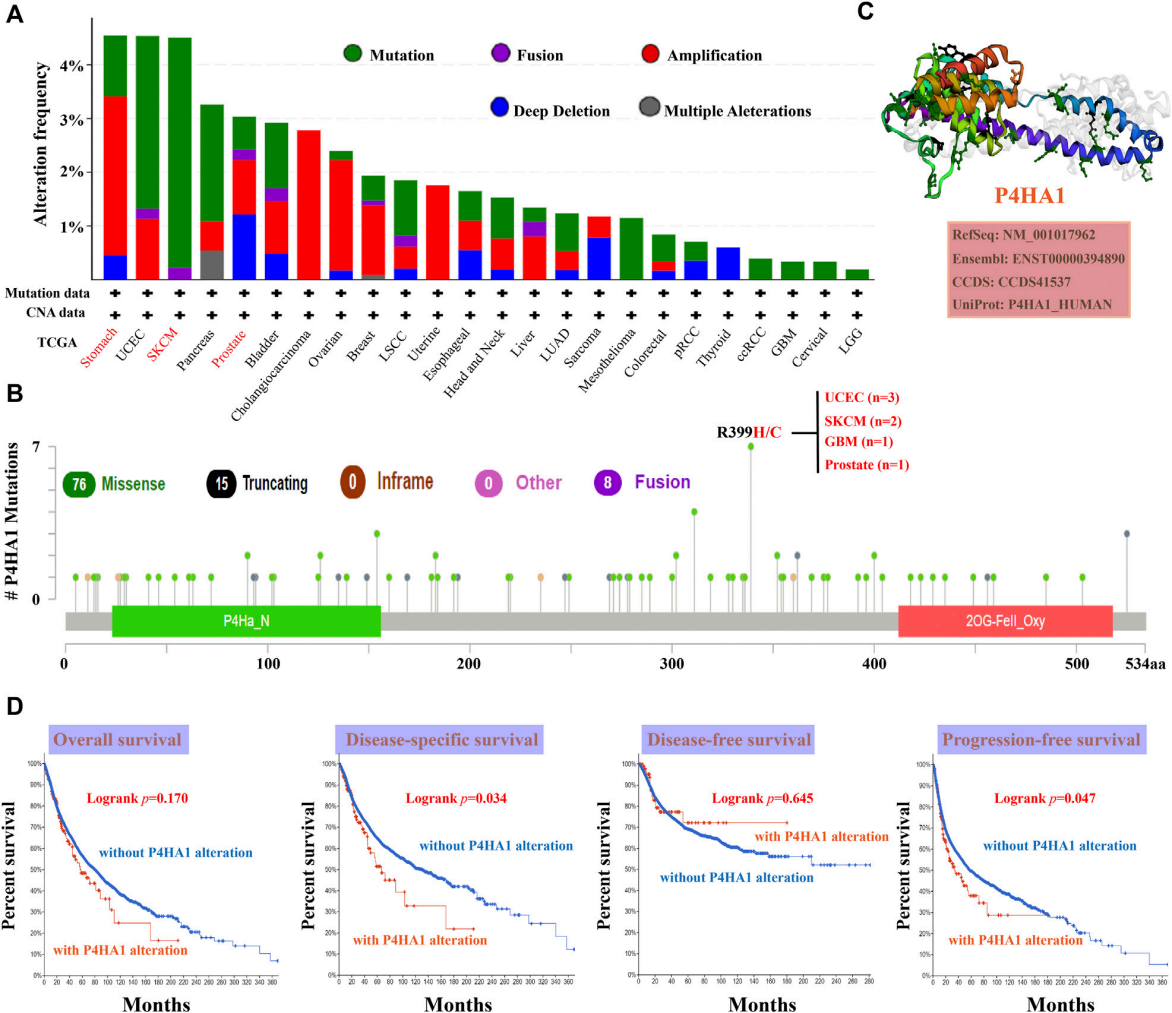
Mutation characteristics of P4HA1 in distinct tumors of TCGA. **(A,B)** cBioPortal tool (http://www.cbioportal.org/) was used to analyze the mutation characteristics of P4HA1 in various cancers. The alteration frequency with mutation type **(A)** and mutation site **(B)** is exhibited. **(C)** 3D structure of P4HA1 was displayed. **(D)** cBioPortal tool was used to analyze the potential correlation between mutation status and OS, disease-specific survival, DFS, and progression-free survival (PFS) of distinct cancers of TCGA.

cBioPortal (https://www.cbioportal.org/) was used to explore the genetic alterations in P4HA1 (Cerami et al., 2012; Gao et al., 2013). As shown in Figure 3A, “mutation” (>4%) was the primary alteration in SKCM patients. “Amplification” was the primary alteration in patients with stomach cancer, with a frequency of 2%. Notably, prostate cancer cases with genetic alteration of P4HA1 (>1% frequency) had copy number deletions. All the data about sites, types, and numbers of P4HA1 genetic alteration are displayed in Figure 3B. The 3D structure of the P4HA1 protein is presented in Figure 3C. Missense mutations in P4HA1 also occurred frequently. The R399H/C mutation was detected in three cases of UCEC, two cases of SKCM, one case of GBM, and one case of PRAD (Figure 3C).

The relationship between the genetic alteration of P4HA1 and prognosis was evaluated using TCGA data. The results indicated that patients with wild-type P4HA1 had better DSS and PFS than patients with P4HA1 genetic alterations, but there was no association between P4HA1 genetic alteration and OS or DFS.

### DNA Methylation Analysis

DNA methylation, catalyzed by DNA methyltransferase (DNMT), is one of the most significant epigenetic modifications, which plays an essential role in regulating gene expression and maintaining genome structure. Abnormal DNA methylation often occurs in the promoter region of transcription factors, mainly through hypermethylation or hypomethylation. DNA hypermethylation often occurs on tumor suppressors, resulting in the inhibition of transcription, while DNA hypomethylation can activate proto-oncogenes and affect the stability of chromosomes. We evaluated the association between the P4HA1 expression level and DNA methylation across all tumors in TCGA. There were positive correlations between P4HA1 expression and the expression of four DNA methyltransferases (DNMT1, DNMT2, DNMT3A, and DNMT3B) in ACC, CESC, chromophobe RCC, papillary RCC, LGG, PRAD, READ, SKCM, TGCT, THCA, UCEC, and UVM (Supplementary Figure S8). We also used the MEXPRESS approach to evaluate the latent correlation between *P4HA1* methylation and carcinogenesis. We found a negative correlation between P4HA1 expression and DNA methylation in BLCA, SARC, and BRCA, whereas a positive association was observed in TGCT (Supplementary Figure S9).

Moreover, the corresponding methods could be found in Supplementary Material.

### Immune Infiltration Analysis

We analyzed the association between P4HA1 expression and immune cell infiltration in different tumor types. As shown in Supplementary Figure S10, P4HA1 expression was remarkedly correlated with the infiltrating immune cells in most cancers (top three cancers: CESC, clear cell RCC, and LGG). Next, we analyzed the correlation of P4HA1 expression and the StromalScore, ImmuneScore, and ESTIMATEScore in TCGA. The top three tumors, most significantly correlated with the expression of P4HA1, were PCPG, LGG, and COAD for the StromalScore and CESC, UCEC, and LGG for the ImmuneScore and ESTIMATEScore (Supplementary Figure S11A). Furthermore, the P4HA1 expression level was positively associated with the infiltrating StromalScore in clear cell and papillary RCC (Supplementary Figure S11B).

**FIGURE 4:**
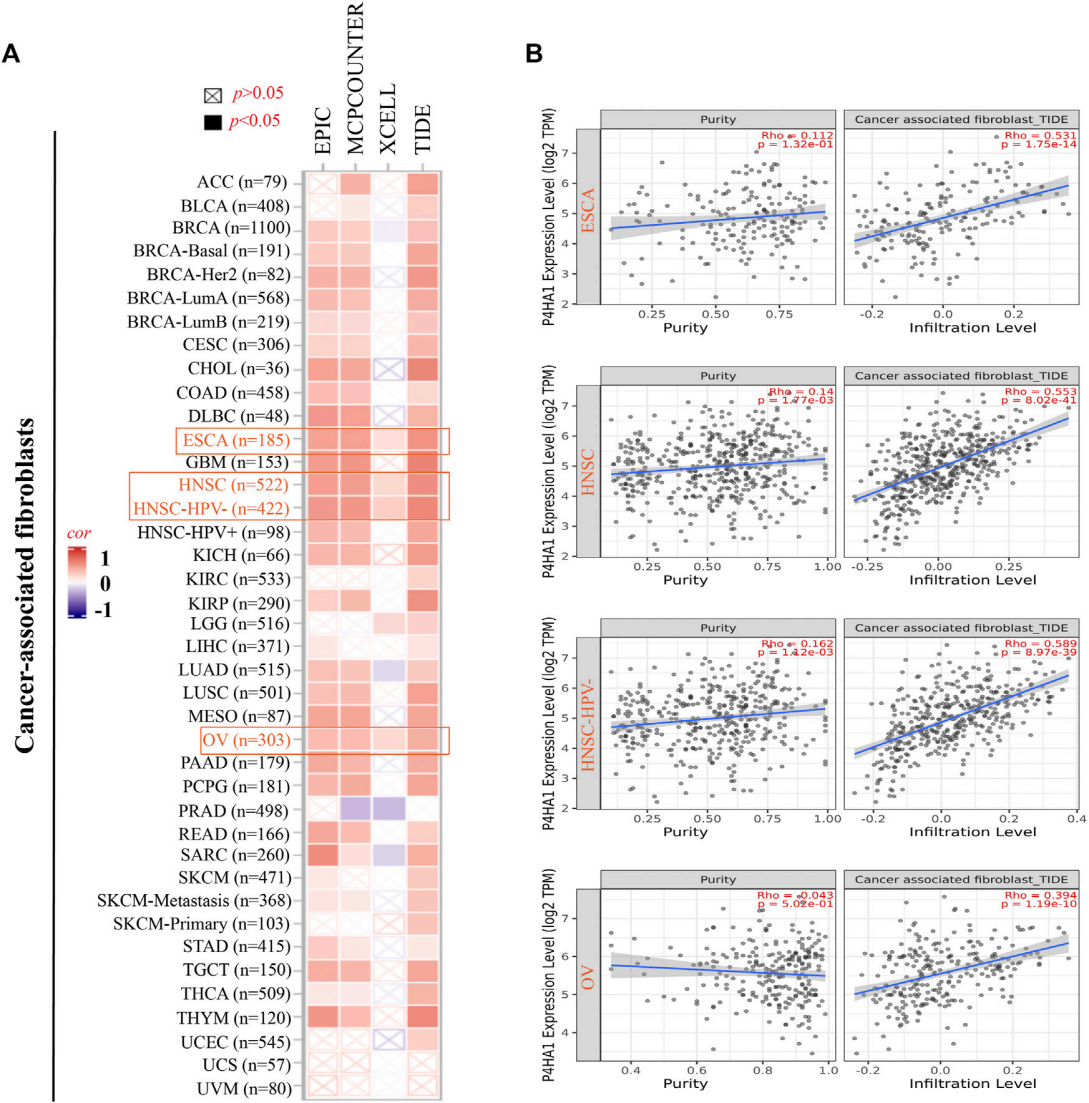
Correlation analysis between the P4HA1 expression level and immune infiltration of cancer-associated fibroblasts (CAFs). **(A,B)** Different algorithms (EPIC, MCPCOUNTER, XCELL, and TIDE) were applied to confirm the potential correlation between the expression level of P4HA1 and the infiltration level of CAFs across distinct types of tumors in TCGA.

**FIGURE 5:**
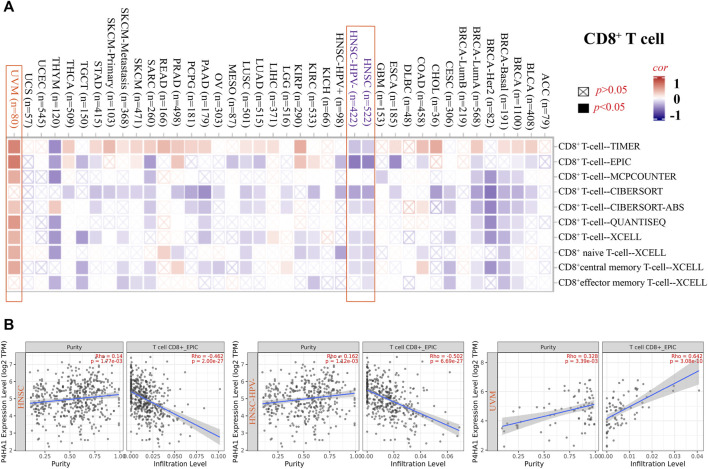
Correlation analysis between SND1 expression and immune infiltration of CD8^+^ T cells. **(A,B)** Different algorithms (TIMER, EPIC, MCPCOUNTER, CIBERSORT, CIBERSORT-ABS, QUANTISEQ, and XCELL) were applied to confirm the potential correlation between the expression level of P4HA1 and the infiltration level of CD8^+^ T cells across different types of tumors in TCGA.

The EPIC algorithms, TIMER, MCPCOUNTER, XCELL, TIDE, TIMER, CIBERSORT, CIBERSORT-ABS, and QUANTISEQ, were used to evaluate the correlation of CAF and CD8^+^ T cell infiltration with P4HA1 gene expression in various cancers. We found a significant positive correlation between P4HA1 expression and CAF infiltration in ESCA, HNSC, HPV-negative HNSC, and OV in TCGA (Figure 4). We also discovered a statistically positive correlation between P4HA1 expression and the estimated infiltration of CD8^+^ T cells in HNSC and HPV-negative HNSC, whereas a statistically negative correlation was observed in UVM (Figure 5) in all or most algorithms.

Furthermore, we carried out a correlation analysis between P4HA1 expression and immune checkpoint gene expression. P4HA1 expression was positively correlated with the expression of TNFSF15, CD80, VTCN1, TNFSF18, and CD200R1 in multiple cancers but negatively correlated with the expression of CD40LG and CD244 in multiple cancers. The associations of P4HA1 expression with LAG3, ICOS, CTLA4, CD48, TNFSF14, and CD27 expression were inconsistent among cancer types (Supplementary Figure S12A). In addition, we investigated the correlation between P4HA1 expression and tumor mutation burden (TMB) and microsatellite instability (MSI). As shown in Supplementary Figures S12B,C, we discovered a positive association between P4HA1 expression and TMB in BRCA, COAD, chromophobe RCC, LUAD, PAAD, PRAD, SKCM, STAD, THYM, and UCEC, but there was a negative correlation between P4HA1 expression and TMB in THCA. Furthermore, P4HA1 expression was positively correlated with MSI in COAD, chromophobe RCC, READ, and STAD but negatively correlated with MSI in BRCA, LUAD, PCPG, and SKCM. Overall, these results have proven that P4HA1 is correlated with tumor immunity, which might explain its influence on the prognosis and survival of cancer patients.

Moreover, the corresponding methods could be found in Supplementary Material.

### Enrichment Analysis of Prolyl 4-Hydroxylase Subunit Alpha 1-Related Genes in Cancers

**FIGURE 6:**
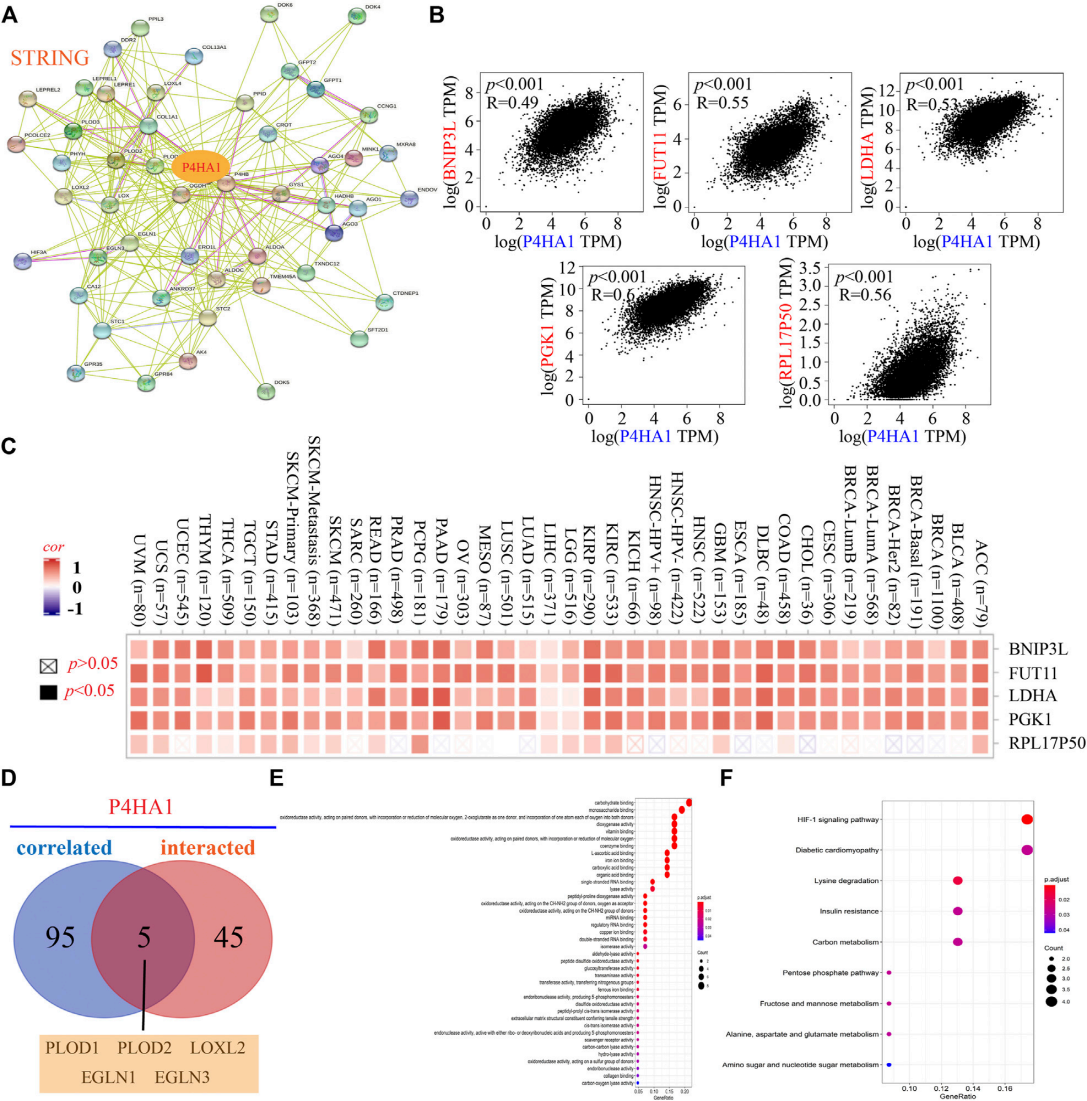
P4HA1-related gene enrichment analysis. **(A)** STRING tool was applied to obtain the P4HA1-binding proteins. **(B)** GEPIA2 was used to obtain the top 100 P4HA1-correlated genes and analyze the expression correlation between P4HA1 and top five related genes (BNIP3L, FUT11, LDHA, PGK1, and RPL17P50). **(C)** Corresponding heatmap analysis showed the correlation between P4HA1 expression and the five related genes (BNIP3L, FUT11, LDHA, PGK1, and RPL17P50) across different types of tumors in TCGA. **(D)** Intersection analysis of the P4HA1-binding and correlated genes was performed. **(E)** Molecular function data of the five genes (PLOD1, PLOD2, LOXL2, EGLN1, and EGLN3) in GO analysis were displayed. **(F)** Five genes (PLOD1, PLOD2, LOXL2, EGLN1, and EGLN3) in KEGG pathway analysis were showed.

To explore the potential mechanism of P4HA1 in tumorigenesis, we screened for genes that bind P4HA1 or affect P4HA1 expression. The STRING database yielded 50 potential P4HA1-binding proteins. The interaction network is shown in Figure 6A. Next, we utilized GEPIA2 to combine the expression data of all tumor types in TCGA and obtained 100 main genes linked to the expression of P4HA1. P4HA1 expression was positively correlated to that of BCL2 interacting protein 3 like (BNIP3L) (R = 0.49, *p* < 0.001), fucosyltransferase 11 (FUT11) (R = 0.55, *p* < 0.001), lactate dehydrogenase A (LDHA) (R = 0.53, *p* < 0.001), phosphoglycerate kinase 1 (PGK1) (R = 0.60, *p* < 0.001), and ribosomal protein L17 pseudogene 50 (RPL17P50) (R = 0.56, *p* < 0.001) (Figure 6B). The corresponding heatmap analysis also indicated a positive correlation between P4HA1 and these five genes (BNIP3L, FUT11, LDHA, PGK1, and RPL17P50) in the majority of 32 types of cancers (Figure 6C). An intersection analysis of P4HA1-related genes and P4HA1-binding components identified five genes: procollagen-lysine, 2-oxoglutarate 5-dioxygenase (PLOD) 1, PLOD2, lysyl oxidase like 2, egl-9 family hypoxia inducible factor (EGLN) 1, and EGLN3 (Figure 6D). This suggested that P4HA1 might play a biological role by interacting with these genes, but the specific molecular mechanism remained to be further verified.

P4HA1-related genes and P4HA1-binding components were then combined to conduct KEGG and GO enrichment analyses. The results of the GO enrichment analysis revealed that most of these genes were linked to the pathways of carbohydrate binding, monosaccharide binding, oxidoreductase activity, and so on (Figure 6E). The KEGG results suggested that “HIF-1 signaling pathway,” “diabetic cardiomyopathy,” “lysine degradation,” and so on might be involved in the effect of P4HA1 on tumorigenesis.

GSEA was conducted to analyze the functional enrichment of the high- and low-P4HA1 expression (Supplementary Figure S13). The KEGG enrichment terms associated with high expression of P4HA1 were primarily related to the cell cycle, oocyte meiosis, and one carbon pool by folate. HALLMARK terms suggested that high expression of P4HA1 was mainly associated with mTORC1 signaling, G2M checkpoint, and glycolysis.

Moreover, the corresponding methods could be found in Supplementary Material.

### Prolyl 4-Hydroxylase Subunit Alpha 1 Promoted the Proliferation, Migration, and Invasion of Renal Cell Carcinoma Cells

**FIGURE 7:**
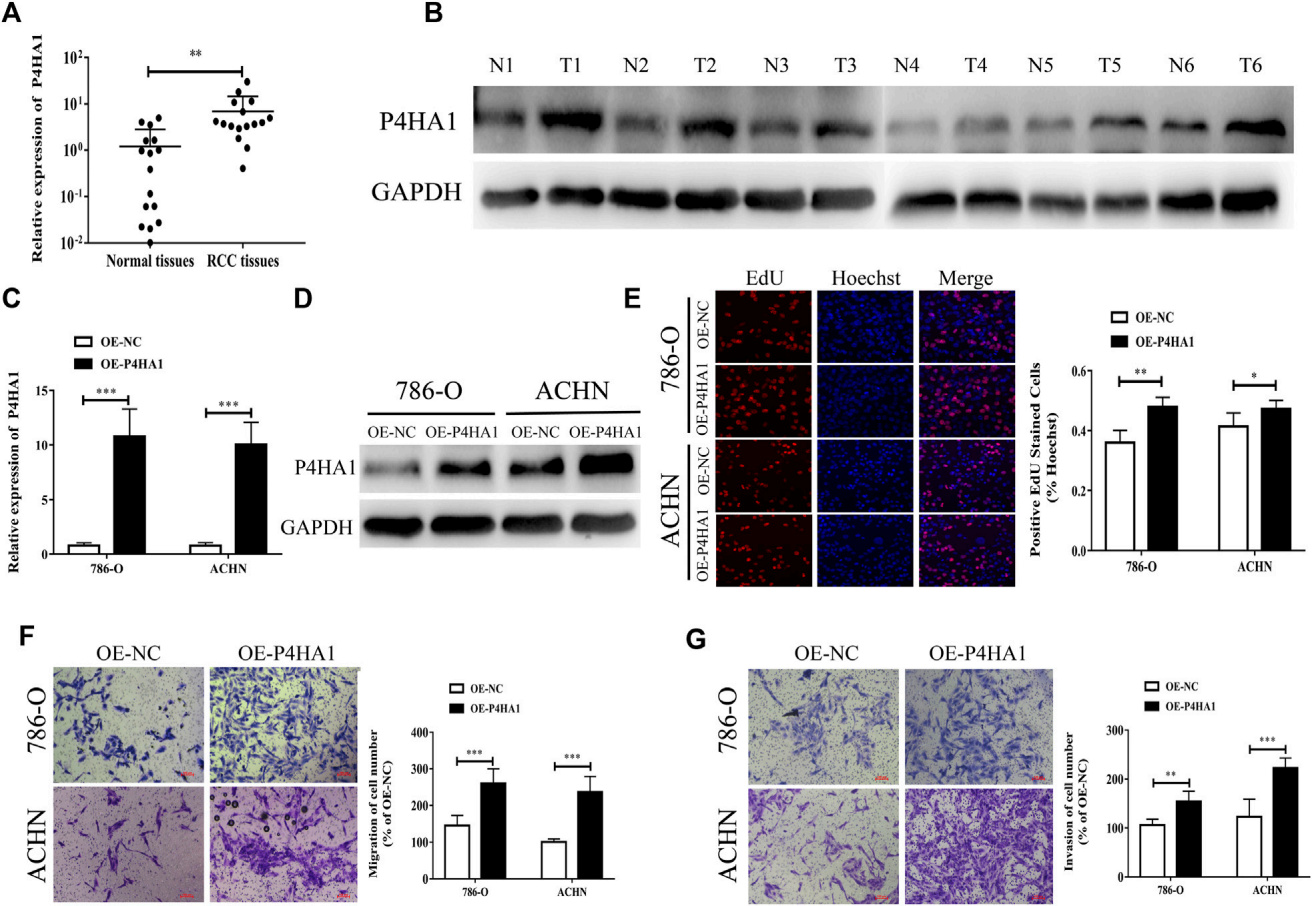
P4HA1 could promote the proliferation, migration, and invasion of RCC. **(A)** QRT-PCR was conducted to detect the mRNA expression level of P4HA1 in RCC tissues. **(B)** Western blot assay was conducted to measure the protein level of P4HA1 in RCC tissues. **(C)** Efficiency of P4HA1 overexpression plasmid (OE-P4HA1) was confirmed by qRT-PCR. **(D)** Efficiency of OE-P4HA1 was confirmed by Western blot. **(E)** EdU assay was conducted to verify the effect of OE-P4HA1 on cell proliferation. **(F,G)** Transwell assay was conducted to verify the effect of OE-P4HA1 on cell migration and invasion.

We found that the expression level of P4HA1 mRNA and protein in KIRC was increased, but there was no significant correlation between the prognosis of KIRC patients and P4HA1 expression and even exerted the opposite trend. The expression level of P4HA1 was closely related to the prognosis of KICH and KIRP patients, but its expression was decreased in the KICH tissues, and there was no difference in the KIRP tissues. Therefore, we decided to collect all the RCC tissue specimens for further research. To further confirm the expression of P4HA1 in RCC tissues, qRT-PCR and Western blot assays were performed. P4HA1 was upregulated in the RCC tissues at both the mRNA and protein levels (Figures 7A,B).

We transfected the RCC cells with a P4HA1 overexpression plasmid (OE-P4HA1) and found that OE-P4HA1 remarkedly increased the expression of P4HA1 (Figures 7C,D). EdU and Transwell assays were conducted to verify the biological function of P4HA1 in the RCC cells. As shown in Figures 7E–G, overexpression of P4HA1 significantly increased the proliferation, migration, and invasion of RCC cells.

### Prolyl 4-Hydroxylase Subunit Alpha 1 Exerted Regulatory Effects in Renal Cell Carcinoma Progression *Via* Regulating the Epithelial–Mesenchymal Transition

**FIGURE 8:**
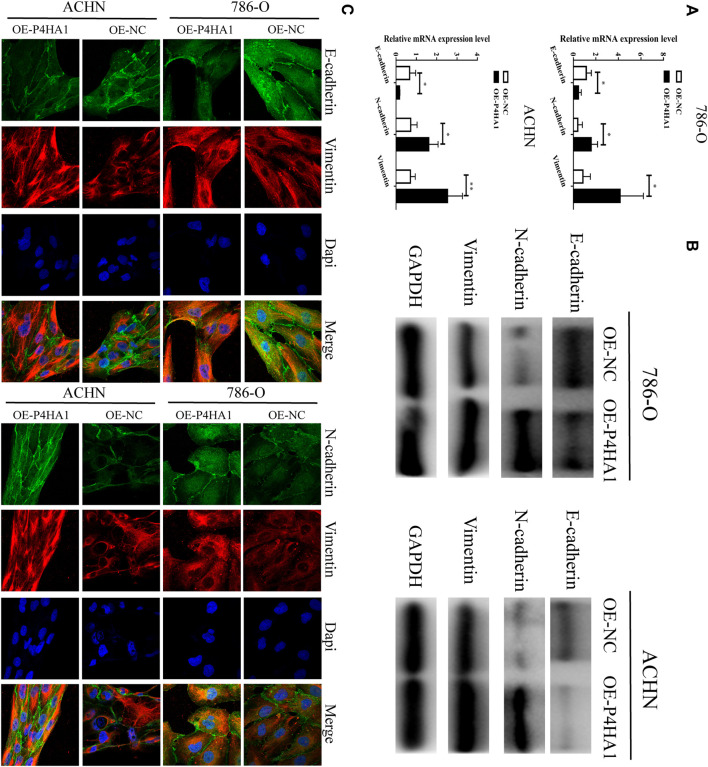
P4HA1 could regulate (epithelial–mesenchymal transformation) the EMT regulatory axis. **(A)** QRT-PCR assay was conducted to detect the mRNA expression of EMT-related genes (E-cadherin, N-cadherin, and Vimentin). **(B)** Western blot assay was conducted to detect the protein expression of EMT-related genes (E-cadherin, N-cadherin, and Vimentin). **(C)** Immunofluorescence confocal was conducted to detect the expression of EMT-related genes.

To explore the potential mechanism of P4HA1 in RCC, we transfected the 786-O and ACHN cells with OE-P4HA1 and assessed the mRNA and protein levels of epithelial–mesenchymal transition (EMT)–related genes by qRT-PCR, Western blot, and immunofluorescence. Transfection of OE-P4HA1 decreased the expression of E-cadherin and increased the expression of N-cadherin and vimentin (Figures 8A–C). These results suggested that P4HA1 might promote RCC progression by promoting EMT. In addition, inhibition of P4HA1 could significantly suppress EMT in the RCC cells (Supplementary Figure S14).

## Discussion

P4HA1 is crucial to the proper three-dimensional folding of the newly synthesized procollagen chains. P4HA1 is also involved in the remodeling of the extracellular matrix (Gilkes et al., 2013). P4HA1 is regarded as an oncogene in several cancer types. In breast cancer, P4HA1 can regulate HIF-1α expression *via* regulating α-KG and succinate levels (Xu, 2019), which promotes chemoresistance by regulating the HIF-1–dependent cancer cell stemness (Xiong et al., 2018). Murugesan et al. showed that P4HA1 may be used for the early detection and therapeutic stratification of breast cancer (Murugesan and Premkumar, 2021). In OV, miR-122 can suppress cell migration, invasion, EMT, and metastasis by targeting *P4HA1* (Duan et al., 2018). P4HA1 can also promote prostate cancer cell growth and tumor progression and is correlated with prostate cancer progression (Chakravarthi et al., 2014). Zhu et al. (2021) showed that the knockdown of P4HA1 can impair the invasion of LN18 and T98G cells by regulating the expression of EMT-related genes. In LUAD, P4HA1 is required for cancer cell growth and invasion (Robinson et al., 2021). Feng et al. (2016) concluded that miR-30e can inhibit the proliferation in hepatoma cells by targeting *P4HA1* mRNA. These studies suggest that P4HA1 is dysregulated in many cancers and essential for biological processes, including cell proliferation, migration, invasion, tumor initiation, and metastasis. Nevertheless, the expression pattern and function of P4HA1 in other tumors, such as RCC, remain unclear. Therefore, we need to comprehensively explore the roles of P4HA1 in human cancers.

We first analyzed the “HomoloGene” and phylogenetic tree data and found that the P4HA1 protein structure was conserved across distinct species. Then, we examined the expression of P4HA1 across 33 different tumor types using the TCGA, CPTAC, GEO, and Oncomine databases. We found that P4HA1 expression was increased in many cancers (BLCA, BRCA, CHOL, COAD, ESCA, GBM, HNSC, clear cell RCC, LUAD, LUSC, PRAD, READ, STAD, THCA, UCEC, DLBCL, LGG, and UCS) compared to adjacent normal tissues. However, P4HA1 was downregulated in the chromophobe RCC and AML tissues. These findings indicated that P4HA1 might play a role in regulating the initiation and progression of tumors.

Next, we analyzed the prognostic significance of P4HA1 using the TCGA and GEO databases. An increased P4HA1 expression was remarkedly associated with poor survival in ACC, BLCA, CESC, chromophobe RCC, papillary RCC, LUAD, LUSC, MESO, PAAD, PCPG, SARC, THCA, and UVM, whereas it was associated with better survival in clear cell RCC. A series of subgroup analyses utilizing several clinical factors was performed to observe different conclusions using the Kaplan–Meier Plotter. Overall, these findings significantly proved that P4HA1 could function as a potential prognostic biomarker in multiple cancer types.

Gene alterations play a vital role in the pathogenesis and progression of tumors (Martinez-Jimenez et al., 2020), and specific alterations can predict treatment response and prognosis (Allegra et al., 2016; Wu et al., 2019). We found that mutation was the primary genetic alteration in P4HA1, and patients with SKCM had the highest alteration frequency of P4HA1. Moreover, we evaluated the correlation between genetic alteration of P4HA1 and prognosis in TCGA and found that patients without altered P4HA1 had better DSS and PFS than those with P4HA1 alterations. Moreover, there was a positive correlation between P4HA1 expression and the expression of four methyltransferases (DNMT1, DNMT2, DNMT3A, and DNMT3B) in many tumor types, including ACC, CESC, chromophobe RCC, papillary RCC, LGG, PRAD, READ, SKCM, TGCT, THCA, UCEC, and UVM. We also found a negative relationship between *P4HA1* methylation and gene expression in BLCA, SARC, and BRCA using MEXPRESS, whereas we observed a positive association in TGCT. However, there is no research on methylation of *P4HA1* in tumors.

Tumor cells can evade immune surveillance, given the proper tumor microenvironment (TME) (Kalluri, 2016). The TME can remarkedly affect therapeutic response and clinical outcome (Quail and Joyce, 2013; Joyce and Fearon, 2015). Emerging evidence has proven that the presence of tumor-infiltrating lymphocytes can be regarded as a potent predictive biomarker in some tumor types (Buisseret et al., 2017; Robbins, 2017). We found that P4HA1 significantly participated in immune cell infiltration in many tumors, especially in CESC, clear cell RCC, and LGG. We also found a positive correlation between P4HA1 expression and the immune score. We used several immune deconvolution methods and found a positive correlation between P4HA1 expression and the infiltration of CAFs in ESCA, HNSC, HPV-negative HNSC, and OV. We also observed a positive correlation between P4HA1 expression and the estimated infiltration value of CD8^+^ T cells in HNSC and HPV-negative HNSC and a negative correlation between P4HA1 expression and infiltration of CD8^+^ T cells in UVM.

Immune checkpoint inhibitors (ICIs) can be incredibly effective in promoting the antitumor immune response (He and Xu, 2020). TMB is regarded as a potential biomarker to predict the response to immune checkpoint blockade (Chan et al., 2019). MSI is also considered a biomarker for the response to ICIs in cancers with distinct clinicopathological characteristics (Sahin et al., 2019). We demonstrated that P4HA1 expression was positively correlated with the expression of TNFSF15, CD80, VTCN1, TNFSF18, and CD200R1 in multiple tumor types but negatively correlated with the expression of CD40LG and CD244. Furthermore, we observed an obvious correlation between P4HA1 expression and TMB and MSI in multiple tumors.

The total protein level of P4HA1 was increased in breast cancer, OV, colon cancer, clear cell RCC, UCEC, and LUAD in the CPTAC dataset. We integrated the information on P4HA1-related genes and P4HA1-binding components across all the tumors using GO and KEGG pathway enrichment analyses and identified the HIF-1 signaling pathway, diabetic cardiomyopathy, lysine degradation, and other pathways that might be related to the effect of P4HA1 on tumorigenesis. We also found that the differentially expressed P4HA1 was primarily correlated to the regulation of cell cycle, oocyte meiosis, and one carbon pool by folate.

In addition, we found that the prognostic value of P4HA1 in clear cell RCC was inconsistent with its mRNA and protein expression. P4HA1 was downregulated in the chromophobe RCC, whereas we observed no significant difference in P4HA1 between papillary RCC and normal tissues. Therefore, we decided to collect all the RCC tissue specimens for further research. To confirm the latent role of P4HA1 in RCC, we conducted qRT-PCR and Western blot analysis and found that P4HA1 was upregulated in the RCC tissues at both the mRNA and protein levels. In the survival analysis of P4HA1, the authors found that low P4HA1 expression was interrelated to poor OS prognosis for KIRC, which was inconsistent with the mRNA and protein expression patterns. Unfortunately, we have not collected enough specimens and clinical information to verify the correlation between the expression level of P4HA1 and the prognosis of RCC patients, and we will further explore it in our future research. Mechanistically, P4HA1 promoted RCC proliferation, migration, and invasion by promoting EMT. Zhu et al. (2021) also showed that P4HA1 can promote cell migration and invasion in the GBM cells *via* inducing EMT under hypoxia.

Although we performed a pan-cancer analysis of P4HA1 examining several aspects, there are still limitations to this study. First, we need to expand the sample size and verify the correlation between P4HA1 expression and the prognosis of RCC patients. Second, the exact mechanism of P4HA1 in RCC needs to be further explored. Third, *in vivo* evidence of the oncogenic role of P4HA1 in RCC is lacking.

## Conclusion

Taken together, our data uncovered that the aberrant expression of P4HA1 is correlated with clinical prognosis, immune cell infiltration, DNA methylation, TMB, and MSI in multiple cancer types. In addition, we also showed that P4HA1 might promote RCC proliferation, migration, and invasion by promoting EMT. Our comprehensive pan-cancer analysis suggested that P4HA1 might be a latent prognostic biomarker for clinical diagnosis and assessment of cancers.

## Data Availability

The original contributions presented in the study are included in the article/Supplementary Material. further inquiries can be directed to the corresponding author.
