# Supplementary material for: The Role of P4HA1 in Multiple Cancer Types and its Potential as a Target in Renal Cell Carcinoma
Source: Front Genet. 2022 Jun 23;13:848456. doi: 10.3389/fgene.2022.848456 (PMC9259937; doi:10.3389/fgene.2022.848456)
Supplement: Supplementary file 2 [file Table1.DOCX]

**Table 1: Sequences of primers for qRT-PCR**

| **Name** |  | **Sequence** |
| --- | --- | --- |
| P4HA1 | Forward | 5’- AGTACAGCGACAAAAGATCCAG -3’ |
|  | Reverse | 5’- CTCCAACTCACTCCACTCAGTA -3’ |
| E-cadherin | Forward | 5’- GTACTTGTAATGACACATCTC -3’ |
|  | Reverse | 5’- TGCCAGTTTCTGCATCTTGC -3’ |
| N-cadherin | Forward | 5’- CGAATGGATGAAAGACCCATCC -3’ |
|  | Reverse | 5’- GGAGCCACTGCCTTCATAGTCAA -3’ |
| Vimentin | Forward | 5’- GAAGAGAACTTTGCCGTTGAAG -3’ |
|  | Reverse | 5’- ACGAAGGTGACGAGCCATT -3’ |
| GAPDH | Forward | 5’- GCTTCGGCAGCACATATACTAAAAT-3’ |
|  | Reverse | 5’- CGCTTCACGAATTTGCGTGTCAT -3’ |
