# Supplementary material for: The Role of P4HA1 in Multiple Cancer Types and its Potential as a Target in Renal Cell Carcinoma
Source: Front Genet. 2022 Jun 23;13:848456. doi: 10.3389/fgene.2022.848456 (PMC9259937; doi:10.3389/fgene.2022.848456)
Supplement: Supplementary file 13 [file Table2.DOCX]

| Table S1. Subgroup analysis on the correlation of P4HA1 expression and prognosis of breast cancer cases. | | | | | | |  |  |  |  |
| --- | --- | --- | --- | --- | --- | --- | --- | --- | --- | --- |
|  |  |  |  |  |  |  |  |  |  |  |
| Factor | Subgroup | Sample size | OS | | DMFS | | RFS | | PPS | |
|  |  |  | HR | P | HR | P | HR | P | HR | P |
| **ER status** | ER positive | 3499 | 0.97 | 0.85 | 1.25 | 0.11 | 1.18 | **0.029** | 0.84 | 0.35 |
|  | ER negative | 2168 | 1.49 | **0.019** | 1.38 | **0.015** | 1.43 | **0.00026** | 0.95 | 0.85 |
| **PR status** | PR positive | 1559 | 1.21 | 0.62 | 1.3 | 0.25 | 1.19 | 0.24 | 0.33 | **0.027** |
|  | PR negative | 1989 | 2.18 | **0.0021** | 1.51 | **0.006** | 1.51 | **0.00053** | 0.9 | 0.84 |
| **HER2 status** | HER2 positive | 1273 | 1.21 | 0.31 | 1.12 | 0.51 | 1.48 | **0.00047** | 0.68 | 0.096 |
|  | HER2 negative | 6262 | 1.29 | **0.022** | 1.27 | **0.0065** | 1.35 | **3.20E-07** | 1.02 | 0.88 |
| **Intrinsic subtype** | Basal | 1494 | 2.14 | **0.00012** | 1.82 | **0.00023** | 1.8 | **3.30E-07** | 1.07 | 0.82 |
|  | Luminal A | 3511 | 0.78 | 0.12 | 0.97 | 0.8 | 1.06 | 0.49 | 0.76 | 0.14 |
|  | Luminal B | 2015 | 1.59 | **0.0094** | 1.11 | 0.45 | 1.36 | **0.00064** | 1.15 | 0.5 |
|  | HER2+ | 515 | 0.88 | 0.66 | 0.93 | 0.77 | 1.66 | **0.0051** | 0.71 | 0.37 |
| **Lymph node status** | Lymph node positive | 2153 | 1.3 | 0.11 | 1.39 | **0.011** | 1.38 | **0.00021** | 0.83 | 0.38 |
|  | Lymph node negative | 2829 | 1.11 | 0.53 | 1.37 | **0.013** | 1.26 | **0.0046** | 0.94 | 0.75 |
| **Grade** | Grade 1 | 576 | 0.53 | 0.17 | 1.62 | 0.24 | 0.85 | 0.52 | 0.49 | 0.16 |
|  | Grade 2 | 1795 | 1 | 0.99 | 0.98 | 0.88 | 1.03 | 0.8 | 0.86 | 0.53 |
|  | Grade 3 | 2058 | 1.44 | **0.016** | 1.54 | 0.0013 | 1.48 | **3.80E-05** | 1.14 | 0.48 |
| **TP53 status** | Wild type | 388 | 0.87 | 0.65 | 1.54 | 0.24 | 1.01 | 0.96 | 0.57 | 0.11 |
|  | Mutated | 272 | 1.28 | 0.48 | 1.5 | 0.29 | 1.64 | **0.042** | 0.85 | 0.71 |
| **Pietenpol subtype** | Basal-like 1 | 418 | 2.32 | **0.036** | 1.66 | 0.081 | 1.43 | 0.1 | 0.86 | 0.81 |
|  | Basal-like 2 | 165 | 1.37 | 0.53 | 1.95 | 0.088 | 3.2 | **0.00034** | NA | NA |
|  | immunomodulatory | 462 | 1.92 | 0.11 | 1.34 | 0.3 | 1.36 | 0.18 | 1.21 | 0.72 |
|  | Mesenchymal | 382 | 1.97 | **0.044** | 1.2 | 0.55 | 1.7 | **0.0089** | 0.72 | 0.45 |
|  | Mesenchymal stem-like | 201 | 2.67 | 0.063 | 2.7 | 0.06 | 1.17 | 0.66 | NA | NA |
|  | Luminal androgen receptor | 413 | 0.62 | 0.12 | 1.04 | 0.9 | 1.19 | 0.37 | 0.59 | 0.22 |
|  |  |  |  |  |  |  |  |  |  |  |
| HR, hazard ratio; OS, overall survival; RFS, relapse free survival; DMFS, distant metastasis free survival; ER, Estrogen receptor; PR, Progesterone receptor; HER2, | | | | | | | | | | |
| human epidermal growth factor receptor-2; TP53, Tumor Protein P53; NA, not available data; P value less than 0.05 is shown in bold. | | | | | | | | | | |
