# Supplementary material for: The Role of P4HA1 in Multiple Cancer Types and its Potential as a Target in Renal Cell Carcinoma
Source: Front Genet. 2022 Jun 23;13:848456. doi: 10.3389/fgene.2022.848456 (PMC9259937; doi:10.3389/fgene.2022.848456)
Supplement: Supplementary file 14 [file Table5.DOCX]

| Table S4. Subgroup analysis on the correlation of P4HA1 expression and prognosis of gastric cancer cases. | | | | | | |  |  |
| --- | --- | --- | --- | --- | --- | --- | --- | --- |
|  |  |  |  |  |  |  |  |  |
| Factor | Subgroup | Sample size | OS | | FP | | PPS | |
|  |  |  | HR | P | HR | P | HR | P |
| **Stage** | Stage 1 | 69 | 0.44 | 0.13 | 0.74 | 0.61 | 0 | 0.04 |
|  | Stage 2 | 145 | 0.92 | 0.8 | 0.82 | 0.53 | 0.93 | 0.82 |
|  | Stage 3 | 319 | 0.73 | **0.028** | 0.9 | 0.57 | 0.6 | **0.019** |
|  | Stage 4 | 152 | 0.79 | 0.23 | 0.96 | 0.84 | 0.67 | 0.084 |
| **Stage T** | T1 | 14 | NA | NA | NA | NA | NA | NA |
|  | T2 | 253 | 0.97 | 0.9 | 0.91 | 0.66 | 0.95 | 0.8 |
|  | T3 | 208 | 0.83 | 0.29 | 0.88 | 0.47 | 0.57 | **0.0046** |
|  | T4 | 39 | 0.57 | 0.19 | 0.68 | 0.32 | 0.47 | 0.12 |
| **Stage N** | N0 | 76 | 0.5 | 0.13 | 0.5 | 0.13 | 0.44 | 0.21 |
|  | N1 | 232 | 0.72 | 0.12 | 0.75 | 0.16 | 0.7 | 0.13 |
|  | N2 | 129 | 0.82 | 0.39 | 1.02 | 0.93 | 0.7 | 0.14 |
|  | N3 | 76 | 0.58 | **0.042** | 0.6 | 0.065 | 0.63 | 0.11 |
| **Stage M** | M0 | 459 | 0.83 | 0.19 | 0.88 | 0.33 | 0.63 | **0.0023** |
|  | M1 | 58 | 1.01 | 0.98 | 1.22 | 0.51 | 0.88 | 0.72 |
| **Lauren classification** | Instestinal | 336 | 0.64 | **0.0056** | 0.8 | 0.21 | 0.6 | **0.016** |
|  | Diffuse | 248 | 1.03 | 0.87 | 1.14 | 0.45 | 0.73 | 0.11 |
|  | Mixed | 33 | 0.62 | 0.36 | 1.42 | 0.49 | NA | NA |
| **Differentiation** | Poorly | 166 | 0.88 | 0.52 | 1.17 | 0.49 | 0.85 | 0.62 |
|  | Moderately | 67 | 1.45 | 0.26 | 1.59 | 0.14 | 1.42 | 0.44 |
|  | Well | 32 | 0.86 | 0.72 | NA | NA | NA | NA |
| **Gender** | Female | 244 | 0.82 | 0.26 | 0.89 | 0.55 | 0.6 | **0.017** |
|  | Male | 566 | 0.67 | **0.00022** | 0.71 | **0.0056** | 0.61 | **0.00023** |
| **Perforation** | Yes | 4 | NA | NA | NA | NA | NA | NA |
|  | No | 169 | 1.19 | 0.4 | 1.24 | 0.27 | 1.02 | 0.95 |
| **Treatment** | Surgery alone | 393 | 1.09 | 0.55 | 1.13 | 0.38 | 0.72 | **0.038** |
|  | 5-Fu based adjuvant | 157 | 1.33 | 0.1 | 1.38 | 0.063 | 1.2 | 0.31 |
|  | other adjuvant | 80 | 1.05 | 0.92 | 0.91 | 0.8 | 0.86 | 0.74 |
| **HER2** | positive | 424 | 0.91 | 0.47 | 0.91 | 0.56 | 0.67 | **0.023** |
|  | negative | 641 | 0.66 | **0.00035** | 0.76 | **0.039** | 0.61 | **0.00083** |
|  |  |  |  |  |  |  |  |  |
| HR, hazard ratio; OS, overall survival; FP, first progression; PPS, post progression survival; | | | | | | | | |
| HER2, human epidermal growth factor receptor-2; NA, not available data; P value less than 0.05 is shown in bold. | | | | | | | | |
