# Supplementary material for: The Role of P4HA1 in Multiple Cancer Types and its Potential as a Target in Renal Cell Carcinoma
Source: Front Genet. 2022 Jun 23;13:848456. doi: 10.3389/fgene.2022.848456 (PMC9259937; doi:10.3389/fgene.2022.848456)
Supplement: Supplementary file 15 [file Table3.DOCX]

| Table S2. Subgroup analysis on the correlation of P4HA1 expression and prognosis of ovarian cancer cases. | | | | | | | |  |
| --- | --- | --- | --- | --- | --- | --- | --- | --- |
|  |  |  |  |  |  |  |  |  |
| Factor | Subgroup | Sample size | OS | | PFS | | PPS | |
|  |  |  | HR | P | HR | P | HR | P |
| **Histology** | Endometrioid | 62 | 1.56 | 0.62 | 0.9 | 0.83 | NA | NA |
|  | Serous | 1232 | 1.12 | 0.16 | 1.19 | **0.018** | 1.12 | 0.19 |
| **Stage** | Stage 1 | 107 | 1.78 | 0.32 | 1.55 | 0.41 | NA | NA |
|  | Stage 2 | 72 | 0.89 | 0.82 | 1.28 | 0.48 | 0.95 | 0.93 |
|  | Stage 3 | 1079 | 1.07 | 0.39 | 1.16 | 0.054 | 1.07 | 0.45 |
|  | Stage 4 | 189 | 1.4 | 0.068 | 1.45 | **0.049** | 1.59 | **0.038** |
| **Grade** | Grade 1 | 56 | 0.5 | 0.15 | 0.92 | 0.87 | NA | NA |
|  | Grade 2 | 325 | 0.99 | 0.94 | 1.13 | 0.39 | 1.02 | 0.9 |
|  | Grade 3 | 1024 | 1.14 | 0.12 | 1.19 | **0.039** | 1.18 | 0.095 |
|  | Grade 4 | 21 | 0.77 | 0.58 | NA | NA | NA | NA |
| **TP53 mutation** | Wild type | 102 | 0.52 | **0.019** | 0.65 | 0.1 | 0.6 | 0.083 |
|  | Mutated | 516 | 1.11 | 0.38 | 1.38 | **0.0041** | 1.15 | 0.27 |
| **Debulk** | Optimal | 802 | 1.06 | 0.6 | 1.14 | 0.19 | 1.06 | 0.65 |
|  | Suboptimal | 536 | 0.97 | 0.77 | 1.19 | 0.1 | 1.04 | 0.75 |
| **Chemotherapy** | Contains platin | 1438 | 1.16 | **0.041** | 1.26 | **0.00046** | 1.14 | 0.13 |
|  | Contains Taxol | 821 | 1.18 | 0.089 | 1.27 | **0.0064** | 1.14 | 0.21 |
|  | Contains Taxol+platin | 804 | 1.16 | 0.14 | 1.25 | **0.011** | 1.12 | 0.29 |
|  | Contains Avastin | 50 | 0.72 | 0.47 | 0.86 | 0.65 | 0.9 | 0.83 |
|  | Contains Docetaxel | 108 | 0.64 | 0.11 | 0.92 | 0.74 | 0.52 | **0.028** |
|  | Contains Gemcitabine | 135 | 0.89 | 0.54 | 0.82 | 0.31 | 0.78 | 0.25 |
|  | Contains Paclitaxel | 248 | 0.79 | 0.3 | 0.91 | 0.59 | 0.86 | 0.57 |
|  | Contains Topotecan | 119 | 0.91 | 0.64 | 0.98 | 0.94 | 0.88 | 0.53 |
|  |  |  |  |  |  |  |  |  |
| HR, hazard ratio; OS, overall survival; PFS, progress free survival; PPS, post progression survival; TP53, Tumor Protein P53; | | | | | | | | |
| NA, not available data; P value less than 0.05 is shown in bold. | | | | | | | | |
