# Supplementary material for: The Role of P4HA1 in Multiple Cancer Types and its Potential as a Target in Renal Cell Carcinoma
Source: Front Genet. 2022 Jun 23;13:848456. doi: 10.3389/fgene.2022.848456 (PMC9259937; doi:10.3389/fgene.2022.848456)
Supplement: Supplementary file 16 [file Table4.DOCX]

| Table S3. Subgroup analysis on the correlation of P4HA1 expression and prognosis of lung cancer cases. | | | | | | |  |  |
| --- | --- | --- | --- | --- | --- | --- | --- | --- |
|  |  |  |  |  |  |  |  |  |
| Factor | Subgroup | Sample size | OS | | FP | | PPS | |
|  |  |  | HR | P | HR | P | HR | P |
| **Histology** | Adenocarcinoma | 865 | 1.07 | 0.59 | 1.22 | 0.21 | 0.89 | 0.63 |
|  | Squamous cell carcinoma | 675 | 0.98 | 0.89 | 1.13 | 0.65 | 0.69 | 0.47 |
| **Stage** | Stage 1 | 652 | 1.22 | 0.14 | 1.05 | 0.81 | 1.01 | 0.97 |
|  | Stage 2 | 320 | 0.86 | 0.43 | 1.22 | 0.46 | 0.55 | 0.071 |
|  | Stage 3 | 70 | 0.96 | 0.88 | NA | NA | NA | NA |
|  | Stage 4 | 4 | NA | NA | NA | NA | NA | NA |
| **Grade** | Grade I | 202 | 1.37 | 0.084 | 1.28 | 0.27 | 1.31 | 0.28 |
|  | Grade II | 310 | 1.37 | **0.048** | 1.43 | 0.089 | 1.18 | 0.52 |
|  | Grade III | 77 | 1.2 | 0.58 | 1.19 | 0.68 | 0.38 | 0.091 |
| **AJCC stage T** | T1 | 475 | 1.58 | **0.0017** | 2.28 | **0.0019** | 0.97 | 0.91 |
|  | T2 | 686 | 1.23 | 0.066 | 1.31 | 0.075 | 1.08 | 0.68 |
|  | T3 | 99 | 0.99 | 0.96 | 1.35 | 0.55 | NA | NA |
|  | T4 | 48 | 1.01 | 0.97 | NA | NA | NA | NA |
| **AJCC stage N** | N0 | 863 | 1.43 | **0.00086** | 1.37 | 0.058 | 0.83 | 0.37 |
|  | N1 | 296 | 1.7 | **0.001** | 1.78 | **0.015** | 1.12 | 0.68 |
|  | N2 | 113 | 0.78 | 0.22 | 1.33 | 0.4 | 0.93 | 0.84 |
| **AJCC stage M** | M0 | 818 | 1.47 | **3.00E-04** | 1.14 | 0.6 | 0.95 | 0.88 |
|  | M1 | 10 | NA | NA | NA | NA | NA | NA |
| **Gender** | Female | 817 | 1.19 | 0.14 | 1.32 | 0.057 | 0.88 | 0.5 |
|  | Male | 1387 | 1.15 | 0.074 | 1.14 | 0.32 | 1.04 | 0.81 |
| **Smoking history** | Exclude those never smoked | 970 | 1.19 | 0.098 | 1.11 | 0.39 | 1.1 | 0.52 |
|  | Only those never smoked | 247 | 1.58 | 0.11 | 1.39 | 0.18 | 0.96 | 0.91 |
| **Surgery success** | Only surgical margins negative | 730 | 1.23 | **0.07** | 1.26 | 0.073 | 1 | 1 |
| **Chemotherapy** | Yes | 178 | 0.86 | 0.48 | 1.39 | 0.12 | 0.89 | 0.62 |
|  | No | 317 | 1.24 | 0.21 | 1.17 | 0.44 | 1.3 | 0.28 |
| **Radiotherapy** | Yes | 73 | 0.85 | 0.56 | 0.93 | 0.78 | 0.8 | 0.47 |
|  | No | 276 | 1.25 | 0.23 | 1.34 | 0.14 | 1.4 | 0.13 |
|  |  |  |  |  |  |  |  |  |
| HR, hazard ratio; AJCC，American Joint Committee on Cancer; OS, overall survival; FP, first progression; | | | | | | | | |
| PPS, post progression survival; NA, not available data; P value less than 0.05 is shown in bold. | | | | | | | | |
