# Supplementary material for: The Role of P4HA1 in Multiple Cancer Types and its Potential as a Target in Renal Cell Carcinoma
Source: Front Genet. 2022 Jun 23;13:848456. doi: 10.3389/fgene.2022.848456 (PMC9259937; doi:10.3389/fgene.2022.848456)
Supplement: Supplementary file 18 [file Table6.DOCX]

| Table S5. Subgroup analysis on the correlation of P4HA1 expression and prognosis of liver cancer cases. | | | | | | |  |  |  |  |
| --- | --- | --- | --- | --- | --- | --- | --- | --- | --- | --- |
|  |  |  |  |  |  |  |  |  |  |  |
| Factor | Subgroup | Sample size | OS | | PFS | | RFS | | DDS | |
|  |  |  | HR | P | HR | P | HR | P | HR | P |
| **Stage** | Stage 1 | 171 | 0.94 | 0.85 | 0.79 | 0.36 | 1 | 0.99 | 0.75 | 0.21 |
|  | Stage 2 | 86 | 0.63 | 0.25 | 0.85 | 0.58 | 0.93 | 0.83 | 0.62 | 0.39 |
|  | Stage 3 | 85 | 1.08 | 0.81 | 0.63 | 0.095 | 0.66 | 0.17 | 0.72 | 0.36 |
|  | Stage 4 | 4 | NA | NA | NA | NA | NA | NA | NA | NA |
| **Grade** | Grade 1 | 55 | 0.73 | 0.6 | 1.51 | 0.3 | 1.64 | 0.33 | 0.73 | 0.6 |
|  | Grade 2 | 177 | 0.78 | 0.34 | 0.72 | 0.13 | 0.75 | 0.24 | 0.49 | **0.041** |
|  | Grade 3 | 122 | 0.85 | 0.59 | 0.68 | 0.13 | 0.8 | 0.41 | 1 | 1 |
|  | Grade 4 | 12 | NA | NA | NA | NA | NA | NA | NA | NA |
| **AJCC_T** | T1 | 181 | 0.91 | 0.74 | 0.77 | 0.3 | 0.94 | 0.8 | 0.79 | 0.57 |
|  | T2 | 94 | 0.67 | 0.29 | 0.77 | 0.36 | 0.82 | 0.53 | 0.71 | 0.48 |
|  | T3 | 80 | 0.8 | 0.46 | 0.69 | 0.19 | 0.73 | 0.31 | 0.71 | 0.35 |
|  | T4 | 13 | NA | NA | NA | NA | NA | NA | NA | NA |
| **Vascular invasion** | None | 205 | 0.83 | 0.47 | 0.95 | 0.82 | 0.93 | 0.77 | 0.9 | 0.77 |
|  | Micro | 93 | 0.76 | 0.49 | 0.49 | **0.015** | 0.64 | 0.16 | 0.64 | 0.42 |
|  | Macro | 16 | NA | NA | NA | NA | NA | NA | NA | NA |
| **Gender** | Male | 250 | 0.84 | 0.44 | 0.78 | 0.17 | 0.79 | 0.24 | 0.74 | 0.3 |
|  | Female | 121 | 1.48 | 0.17 | 1.43 | 0.17 | 1.36 | 0.3 | 1.73 | 0.13 |
| **Race** | White | 184 | 0.95 | 0.84 | 0.86 | 0.46 | 1.1 | 0.67 | 0.9 | 0.72 |
|  | Bkack | 17 | NA | NA | NA | NA | NA | NA | NA | NA |
|  | Asian | 158 | 0.7 | 0.24 | 0.7 | 0.13 | 0.65 | 0.097 | 0.7 | 0.37 |
| **Sorafenib treatment** | Treated | 30 | 0.81 | 0.7 | 0.92 | 0.83 | 1.43 | 0.43 | 0.76 | 0.61 |
| **Alcohol consumption** | Yes | 117 | 0.89 | 0.73 | 0.72 | 0.2 | 0.66 | 0.16 | 0.84 | 0.64 |
|  | none | 205 | 1.07 | 0.77 | 0.79 | 0.24 | 0.94 | 0.78 | 0.88 | 0.69 |
| **Hepatitis virus** | Yes | 153 | 0.88 | 0.7 | 0.81 | 0.37 | 1.17 | 0.54 | 0.88 | 0.75 |
|  | none | 169 | 1.05 | 0.85 | 0.78 | 0.26 | 0.79 | 0.36 | 0.98 | 0.94 |
|  |  |  |  |  |  |  |  |  |  |  |
| HR, hazard ratio; AJCC，American Joint Committee on Cancer; OS, overall survival; PFS, progress free survival; | | | | | | | | | | |
| RFS, relapse free survival; DSS, disease specific surviva; NA, not available data; P value less than 0.05 is shown in bold. | | | | | | | | | | |
