# Supplementary material for: The Role of P4HA1 in Multiple Cancer Types and its Potential as a Target in Renal Cell Carcinoma
Source: Front Genet. 2022 Jun 23;13:848456. doi: 10.3389/fgene.2022.848456 (PMC9259937; doi:10.3389/fgene.2022.848456)
Supplement: Supplementary file 21 [file DataSheet1.DOCX]

**Supplementary data**

**1. Supplementary materials and methods**

**1.1 Gene mapping and protein structure analysis**

The genomic location information of the P4HA1 was acquired by utilizing UCSC genome browser (http://genome.ucsc.edu/)(Kent et al., 2002). We also evaluated the conserved functional domains of P4HA1 proteins in distinct species with the help of "HomoloGene" function of the NCBI (https://www.ncbi.nlm.nih.gov/homologene/). Additionally, the P4HA1 phylogenetic trees in distinct species was calculated by utilizing the constraint-based multiple alignment on-line tool of the NCBI (<https://www.ncbi.nlm.nih.gov/tools/cobalt/>).

**1.2 HPA database analysis**

The expression patterns of P4HA1 in distinct tissues and cell lines were evaluated by the HPA (Human protein atlas) database (<https://www.proteinatlas.org/humanproteome/pathology>). “Low specificity” was defined by “NX (Normalized expression) ≥ 1 in at least one tissue/region/cell type without elevating in any tissue/region/cell type”.

**1.3 Oncomine database analysis**

The Oncomine database(https://www.oncomine.org/resource/main.html) was applied to analyze the distinct expression patterns of P4HA1 between tumor and normal tissues. Several pooled analysis were conducted through at least 10 comparisons.

**1.4 Kaplan-Meier plotter analysis**

We conducted overall survival (OS), survival without distant metastasis (DMFS), survival without recurrence (RFS), survival after progression (PPS), primary progression (FP), disease-specific survival (DSS), and progression-free survival (PFS) on distinct GEO datasets by utilizing Kaplan-Meier plotter to generate corresponding plots. Besides, meta function was utilized to perform meta-analysis on the above P4HA1 survival data in the Stata software.

**1.5 Correlation of P4HA1 and TMB/MSI**

We utilized Sanger tool (http://sangerbox.com/Tool)(Bonneville et al., 2017) to investigate the latent correlation between P4HA1 expression and TMB (tumor mutational burden) or MSI (microsatellite instability) in each available tumor and then conducted searman rank correlation test.

**1.6 DNA methylation analysis**

We analyzed the correlation between gene expression and the expression of four methyltransferases (DNMT1: red, DNMT2: blue, DNMT3A: green, and DNMT3B: purple) by Sanger tool. The MEXPRESS web (https://mexpress.be/) was utilized to evaluate the DNA methylation levels of multiple probes P4HA1 in distinct tumors and acquired beta values for each sample. The promoter region probes were highlighted.

**1.7 Estimate and immune checkpoint gene correlation analysis**

Immune and stromal scores were calculated by utilizing the ESTIMATE (Estimation of STromal and Immune cells in Malignant Tumor tissues with Expression data) algorithm. We also investigated the correlation between P4HA1 and immune checkpoint gene (ICG) expression by Sanger tool. The correlation of gene expression was analyzed using Spearman’s correlation and statistical significance.

**1.8 Gene Set Enrichment Analysis (GSEA)**

To investigate the biological signaling pathway, GSEA was conducted in the highexpression and the low-expression groups compared with the median level of P4HA1 expression respectively. The top 5 terms of KEGG and HALLMARK analysis were displayed. KEGG pathways with significant enrichment results were confirmed on the basis of NES (Net enrichment score), gene ratio, and P value. Gene sets with |NES|>1, NOM p <0.05, and FDR q <0.25 were considered to be enrichment significant(Subramanian et al., 2005).

**2. Supplementary figure legends**

Figure S1. Structural features of P4HA1 in distinct species.

A. Genomic location of P4HA1 (Homo sapiens). B. Conserved domains of P4HA1 protein among distinct species.

Figure S2. Phylogenetic tree of P4HA1.

Constraint-based multiple alignment tool of NCBI was used to obtain the phylogenetic tree of P4HA1 in distinct species.

Figure S3. Expression level of P4HA1 in distinct cells, tissues and plasma in the normal physiological state.

A. The expression of the P4HA1was confirmed in distinct tissues using the consensus datasets of HPA, GTEx and FANTOM5. B. The expression of the P4HA1 was confirmed in immune cells using the consensus dataset of HPA, GTEx and FANTOM5. C. The expression of the P4HA1 was confirmed in blood cells using the consensus dataset of HPA, Monaco and Schmiedel.

Figure S4. Expression level of P4HA1 in distinct tumors and pathological stages.

A. The expression statuses of P4HA1 in ACC, OV, PCCG, SARC, TGCT and THYM in TCGA project were compared with the corresponding normal tissues of the GTEx databases. Expression levels of P4HA1 by different pathological stages of BLCA, BRCA, CHOL, COAD (B); DLBC, ESCA, KIRC, LIHC (C); LUSC, OV, PAAD, READ (D); SKCM, STAD, THCA, UCEC and UCS (E).

Figure S5. Pooled analysis on the P4HA1 expression difference between tumor and normal tissues via the Oncomine database. (A) Brain and CNS cancer; (B) Breast cancer; (C) Colorectal cancer; (D) Head and neck cancer; (E) Kidney cancer; (F) Lung cancer; (G) Pancreatic cancer; (H) Sarcoma.

Figure S6. Correlation between P4HA1 expression level and prognosis of tumors using the Kaplan-Meier plotter.

Kaplan-Meier plotter was used to conduct survival analysis (OS, DMFS, RFS, PPS, PFS, FP and DSS) by the expression level of P4HA1 in BRCA (A), OV (B), lung cancer (C), GC (D), and liver cancer (E) cases.

Figure S7. Meta-analysis on the correlation between P4HA1 expression and cancer prognosis.

We conducted a meta-analysis for pooling of survival analysis by the expression level of P4HA1 in BRCA, OV, lung cancer, GC, and liver cancer cases.

Figure S8. The association between P4HA1 expression and DNA methylation.

Figure S9. Association between P4HA1 DNA methylation and gene expression for the TGCT cases of BLCA, SARC, BRCA and TCGA. MEXPRESS approach was used to analyze the DNA methylation level of P4HA1 of multiple probes.

Figure S10. Correlation of P4HA1 expression with immune infiltration level in different tumors.

Correlation of P4HA1 expression with immune infiltration level in CESC, KIRC, and LGG. P4HA1 expression has significant negative correlation with tumor purity, and significant positive correlation with infiltrating levels of B cell, CD8+ T cell, CD4+ T cell, macrophage, neutrophil, and dendritic cell. *p < 0.05, **p < 0.01, and ***p < 0.001.

Figure S11. Correlation of scores with P4HA1 expression in cancers.

A. Top three cancers by ImmuneScore, StromalScore, and ESTIMATEScore, respectively. Correlation of ImmuneScore and StromalScore. B. Correlation of the estimated proportion of immune and stromal with P4HA1 expression in RCC, and analysis was used by ImmuneScore, StromalScore, and ESTIMATEScore.

Figure S12. Correlations between P4HA1 expression and immunity, including immune marker sets, TMB and MSI in cancers. (A) Correlation between P4HA1 expression and immune marker sets. (B) Radar map of correlation between P4HA1 expression and TMB. (C) Radar map of correlation between P4HA1 expression and MSI.

Figure S13. GSEA for samples with high P4HA1 expression and low expression.

A. The enriched gene sets in KEGG collection by the high P4HA1 expression sample. B. The enriched gene sets in KEGG by samples with low P4HA1 expression. C. Enriched gene sets in HALLMARK collection, the immunologic gene sets, by samples of high P4HA1 expression. D. Enriched gene sets in HALLMARK by the low P4HA1 expression. Each line representing one particular gene set with unique color, and up-regulated genes located in the left approaching the origin of the coordinates, by contrast the down-regulated lay on the right of x-axis. Only gene sets with NOM p < 0.05 and FDR q < 0.06 were considered significant. And only several leading gene sets were displayed in the plot.

Figure S14. Inhibition of P4HA1 could suppress (epithelial mesenchymal transformation) EMT regulatory axis.

A. The efficiency of P4HA1 siRNA (si-P4HA1) was confirmed by qRT-PCR. D. The efficiency of si-P4HA1 was confirmed by western blot. C. Western blot assay was conducted to detect the protein expression of EMT related genes (E-cadherin, N-cadherin and Vimentin).

**3. Supplementary figures**

**Figure S1**


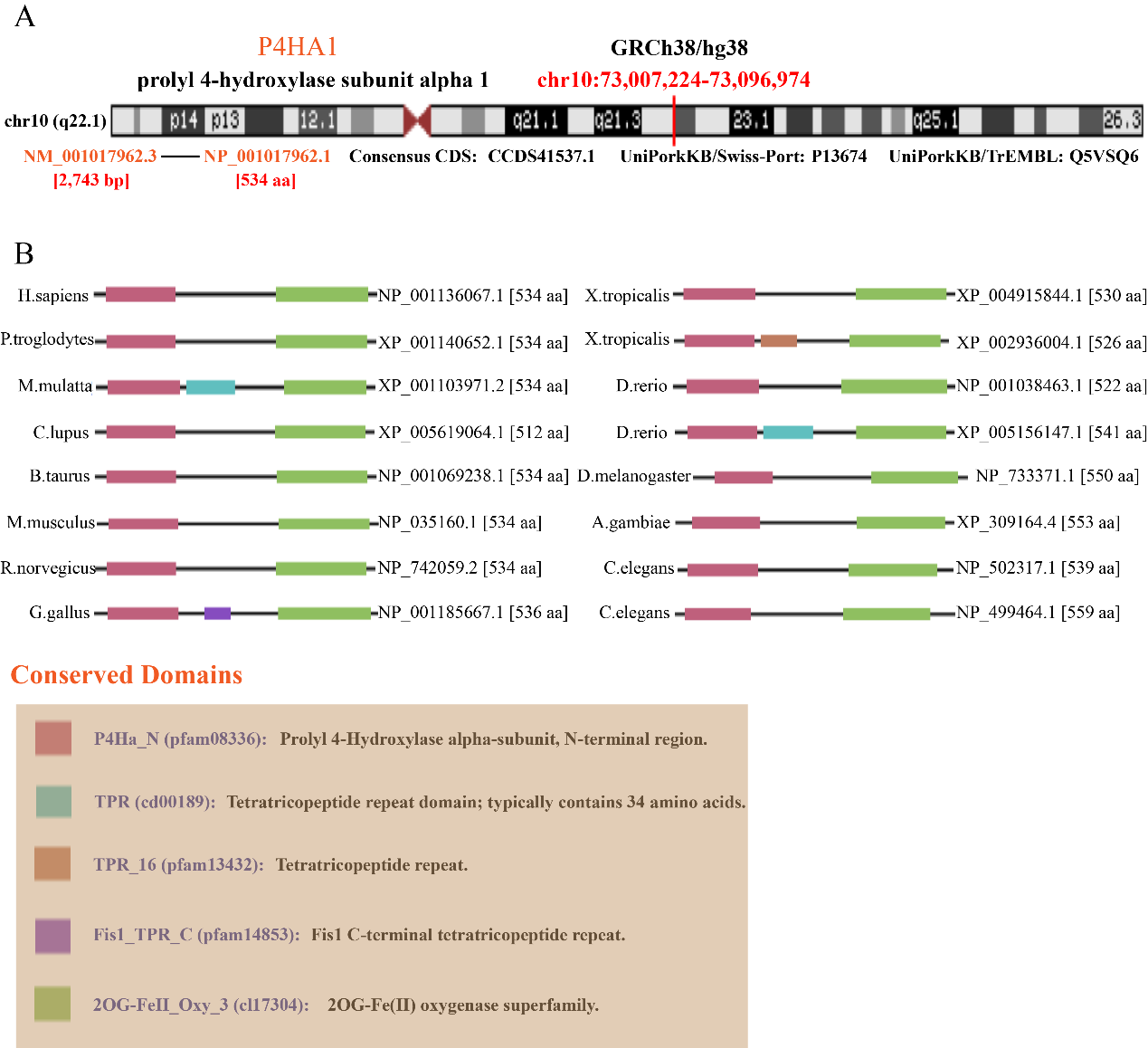


**Figure S2**

**
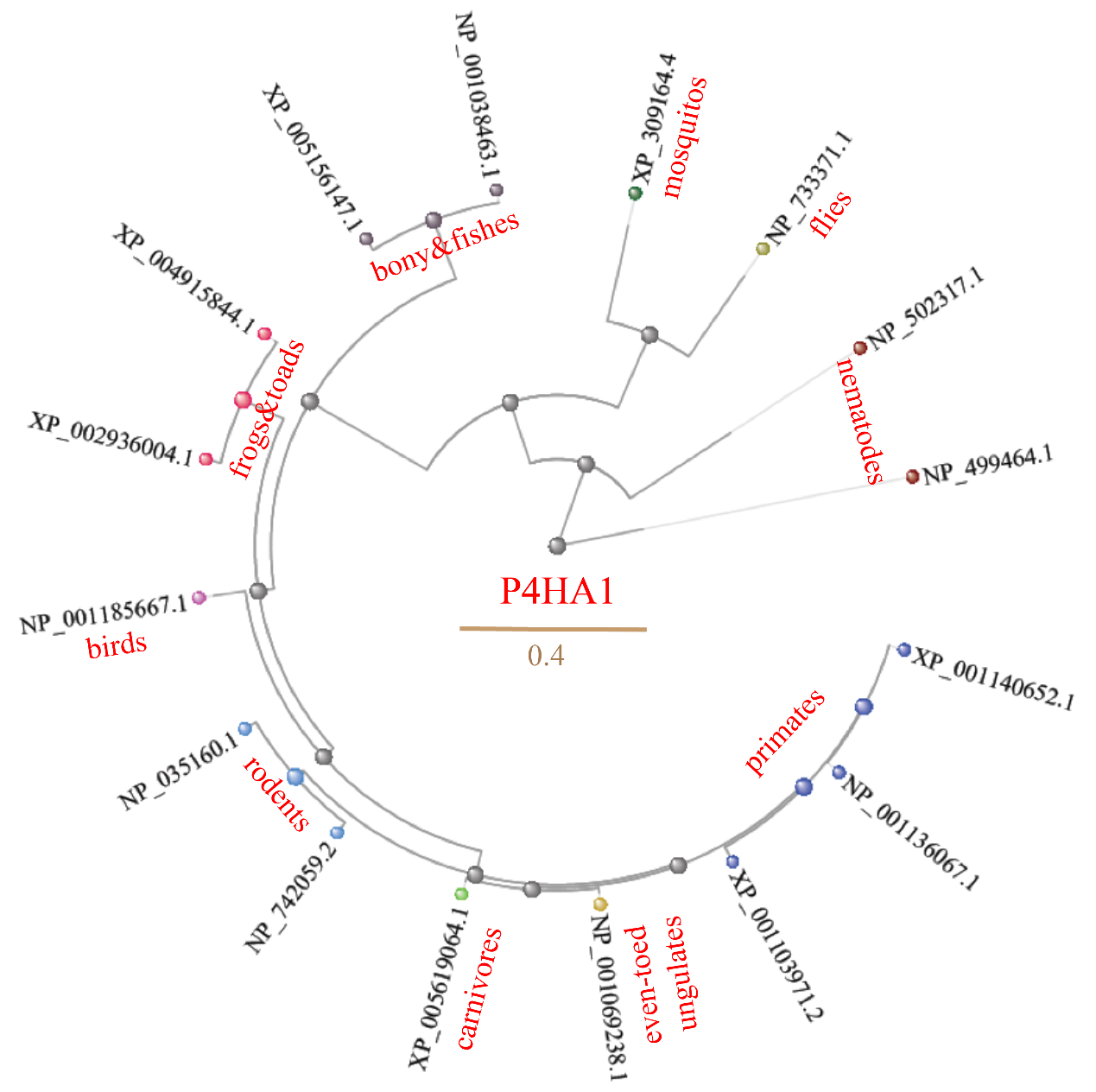
**

**Figure S3**

**
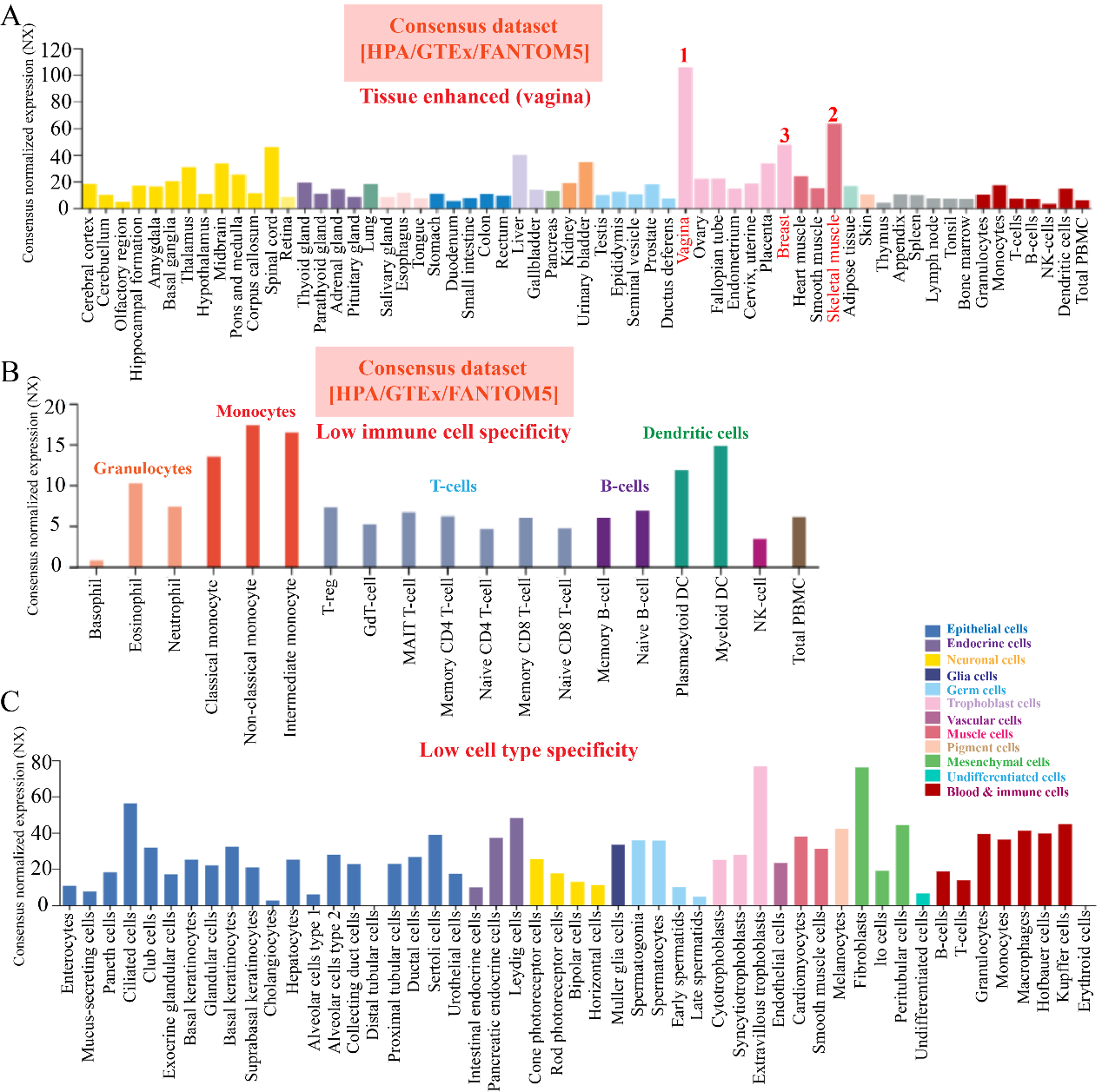
**

**Figure S4**

**
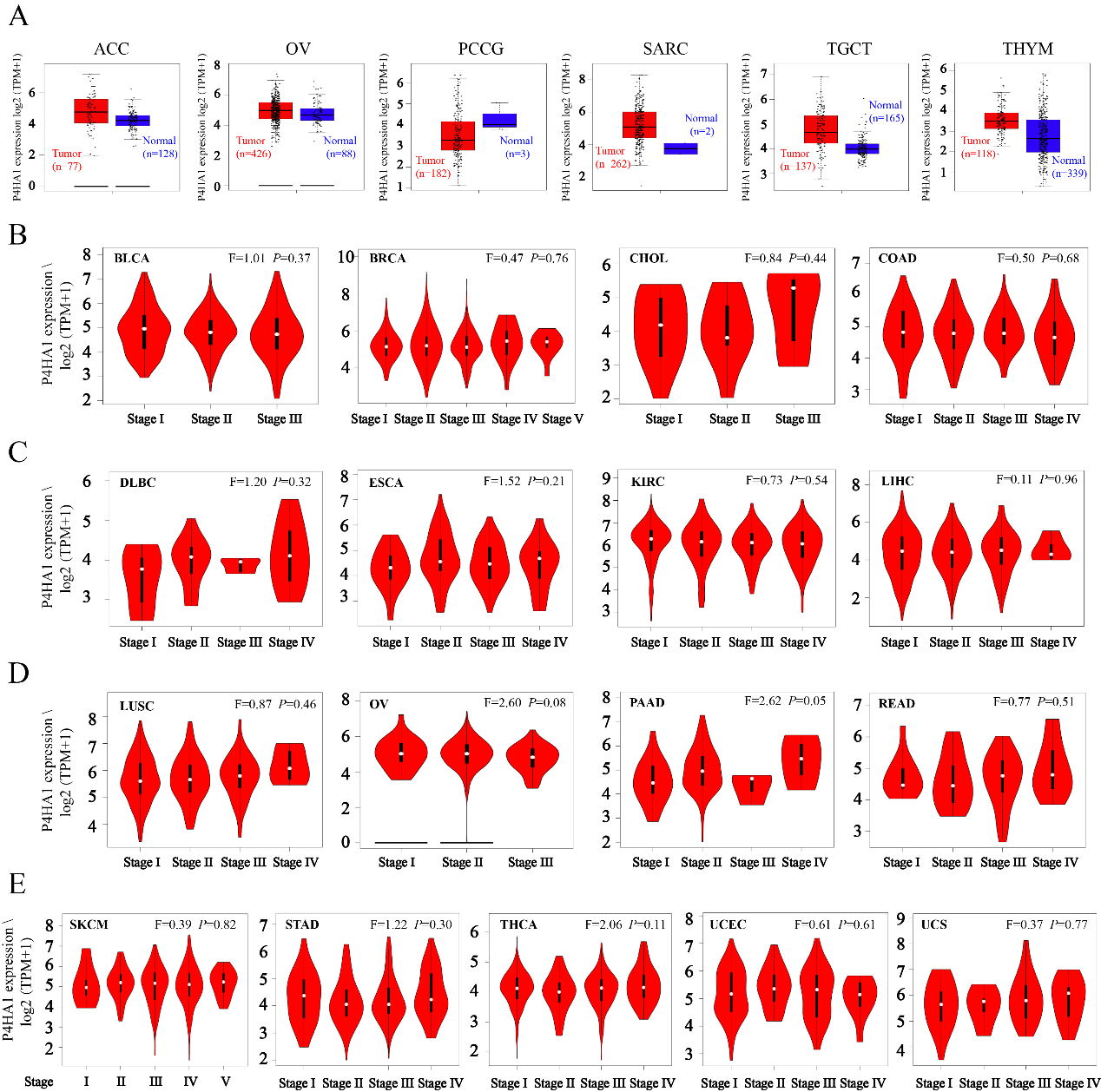
**

**Figure S5**

**
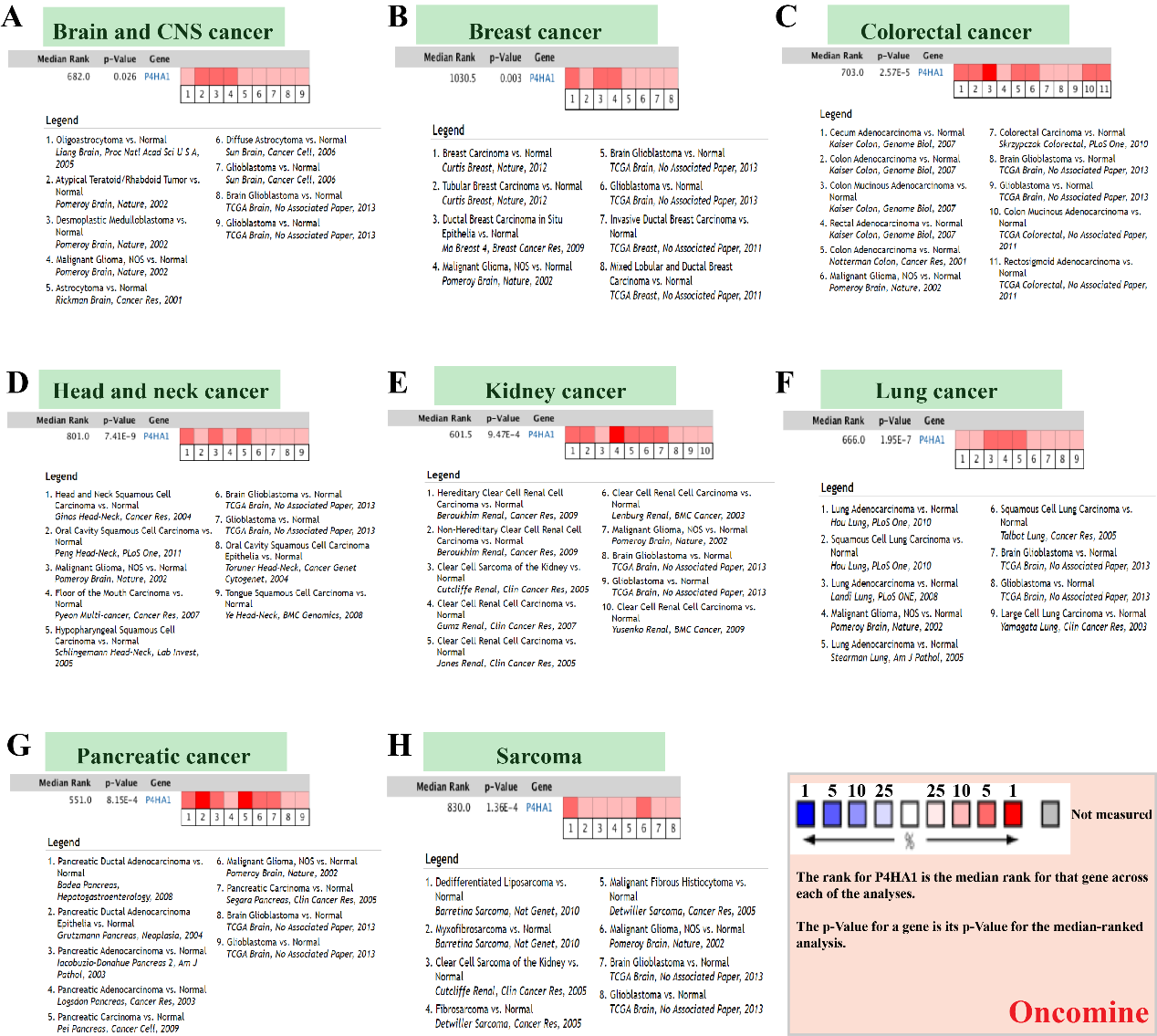
**

**Figure S6**

**
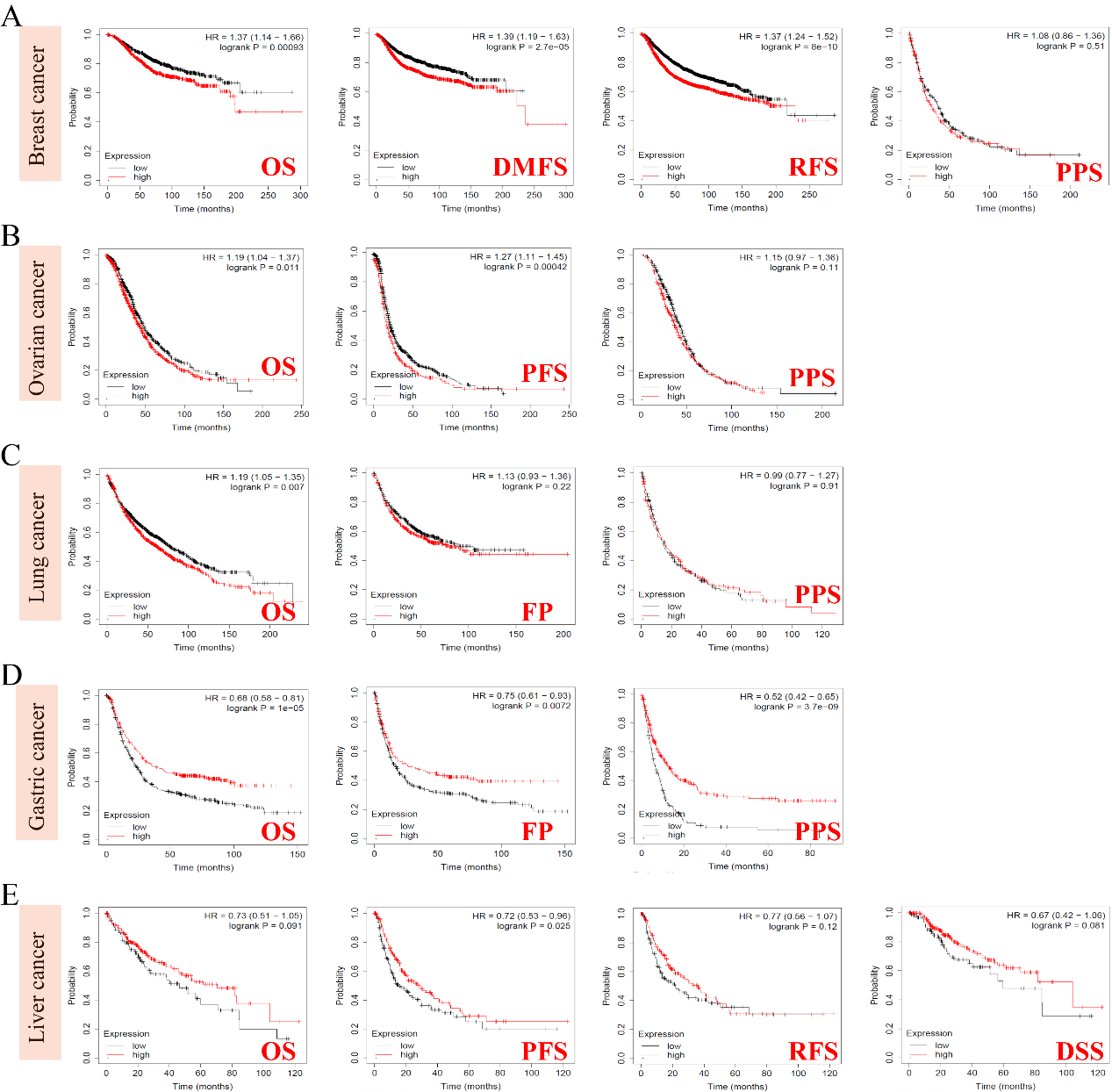
**

**Figure S7**

**
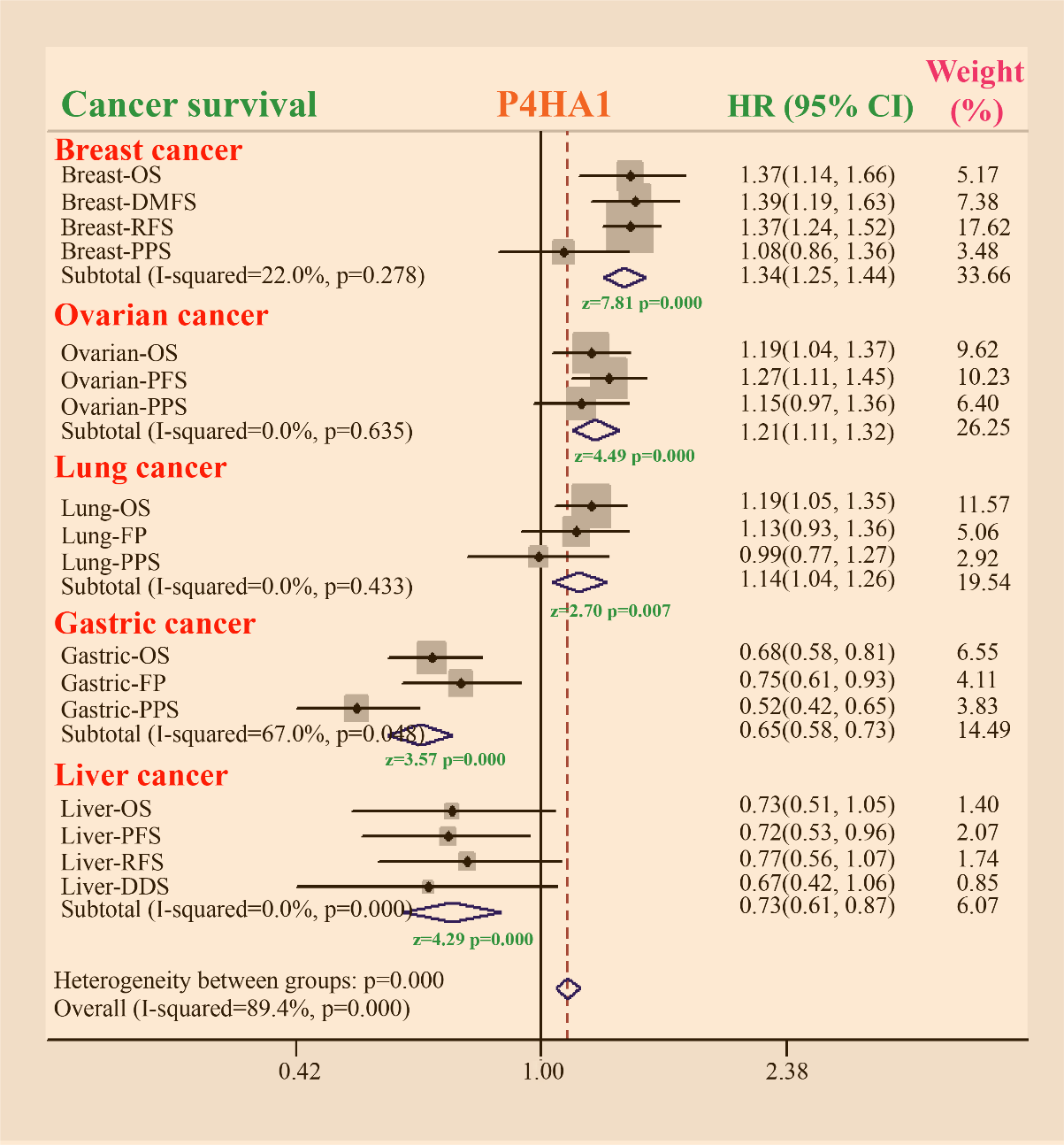
**

**Figure S8**

**
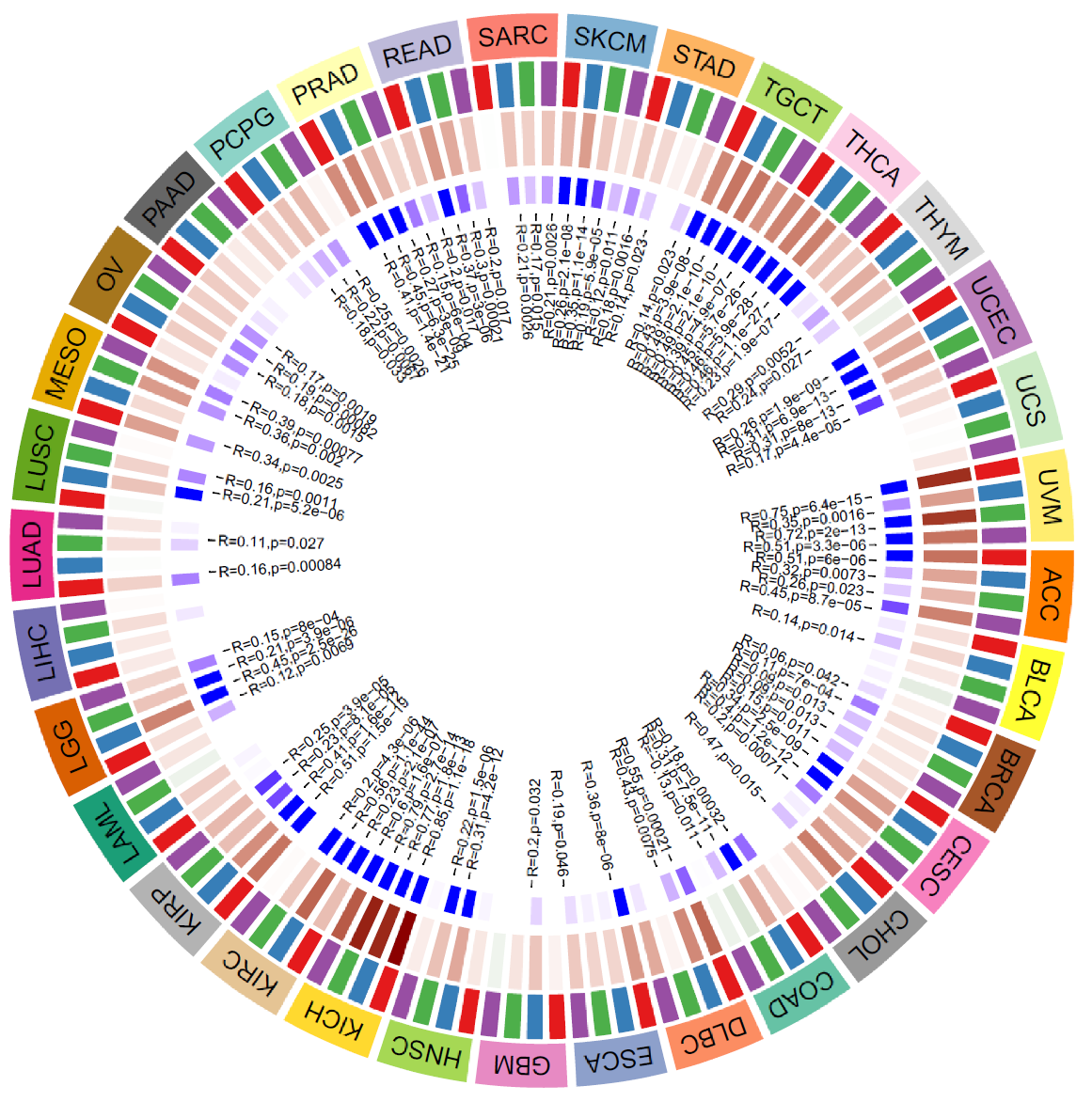
**

**Figure S9**

**
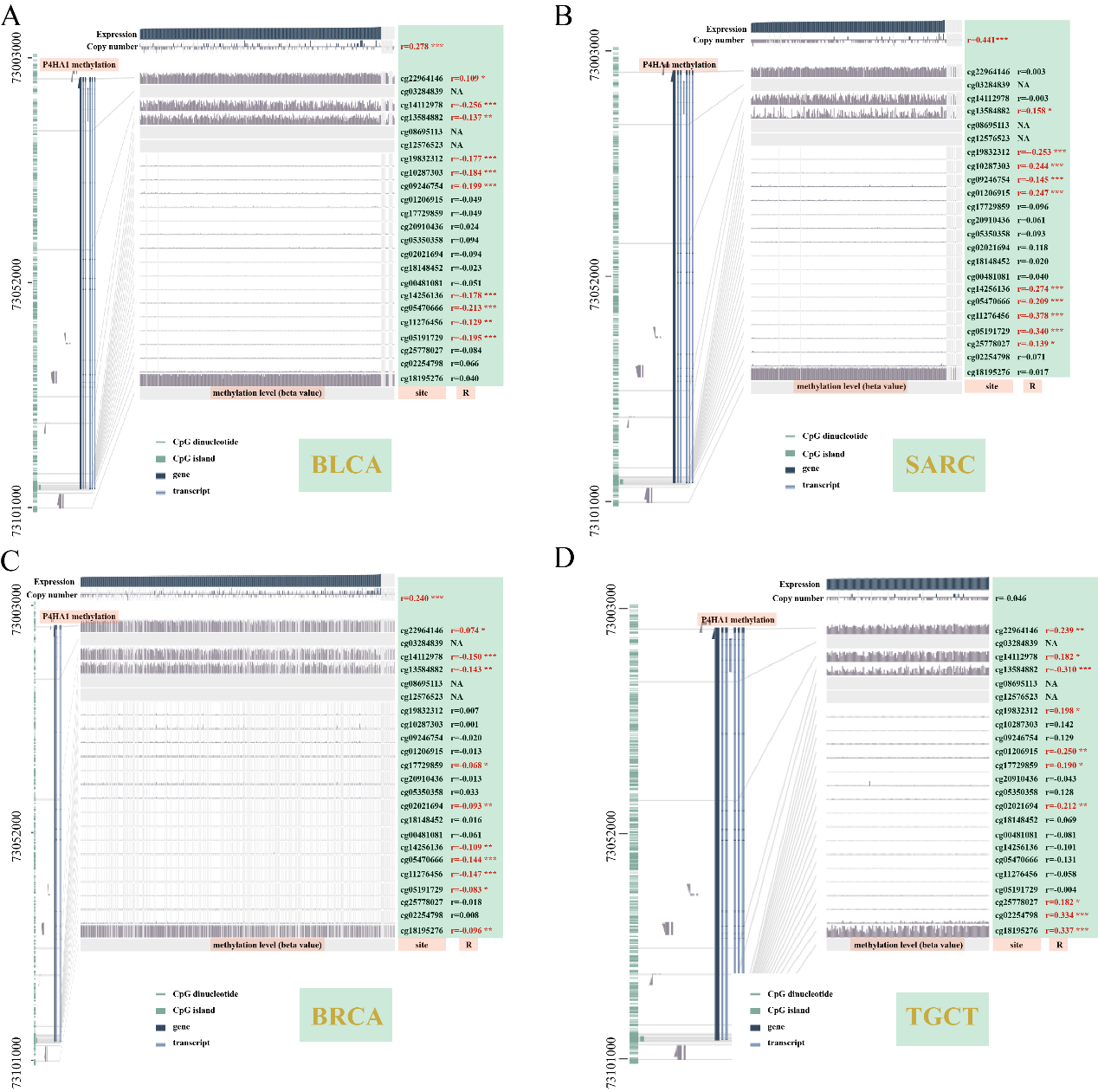
**

**Figure S10**

**
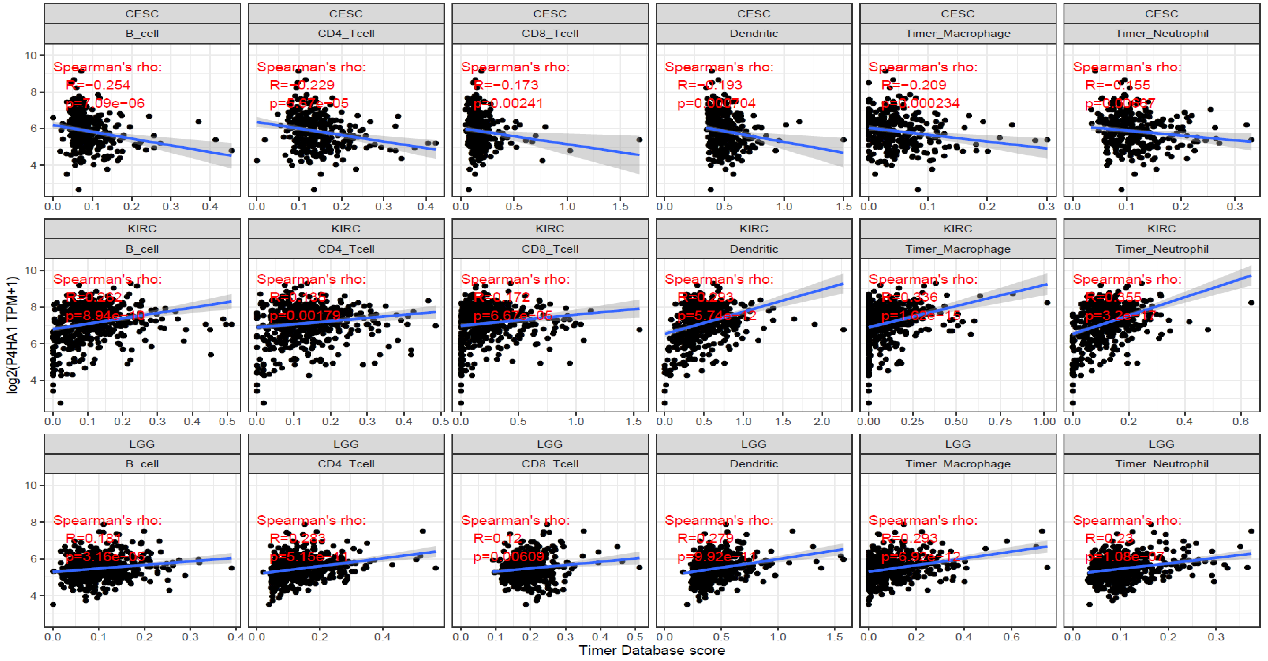
**

**Figure S11**

**
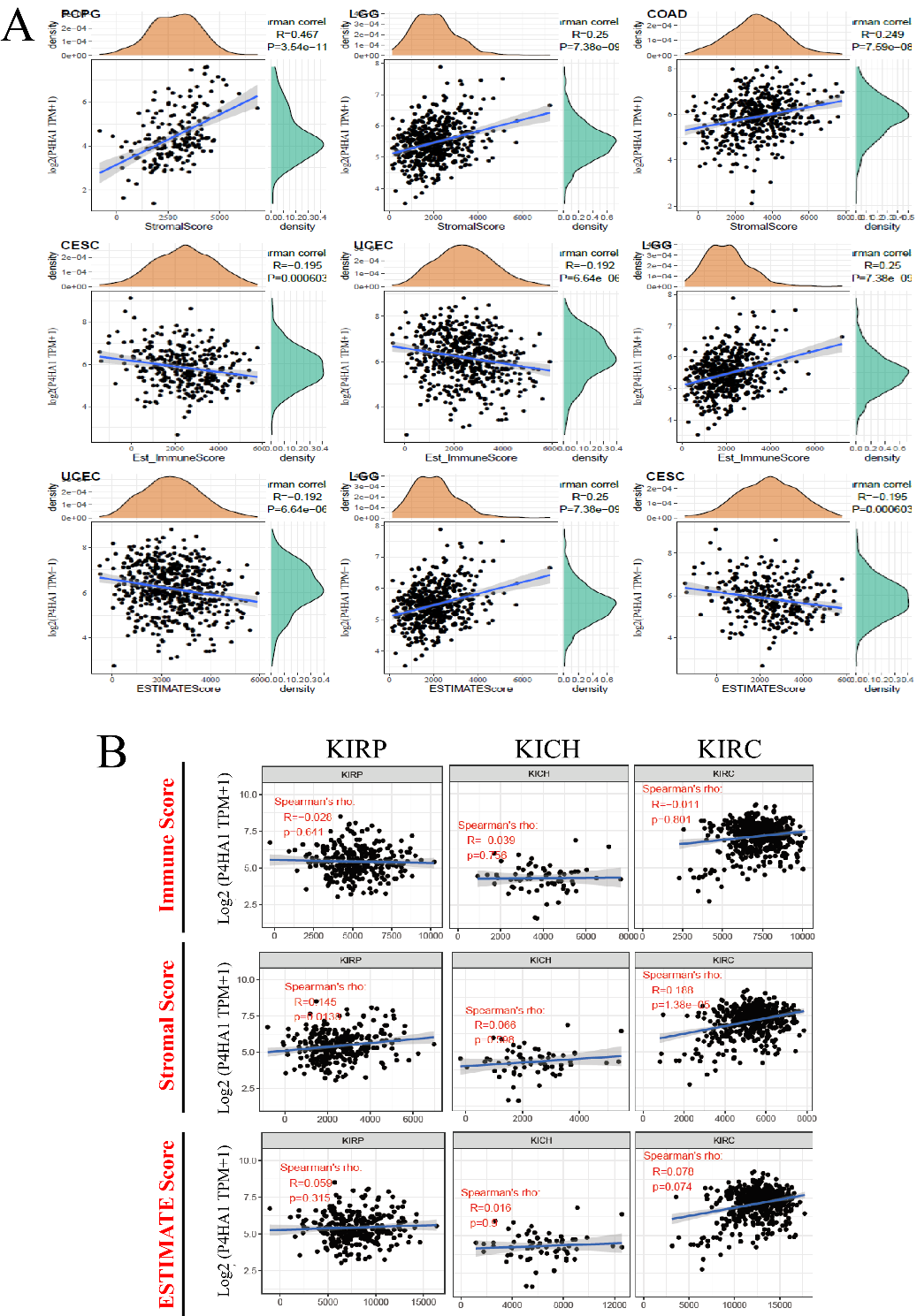
**

**Figure S12**

**
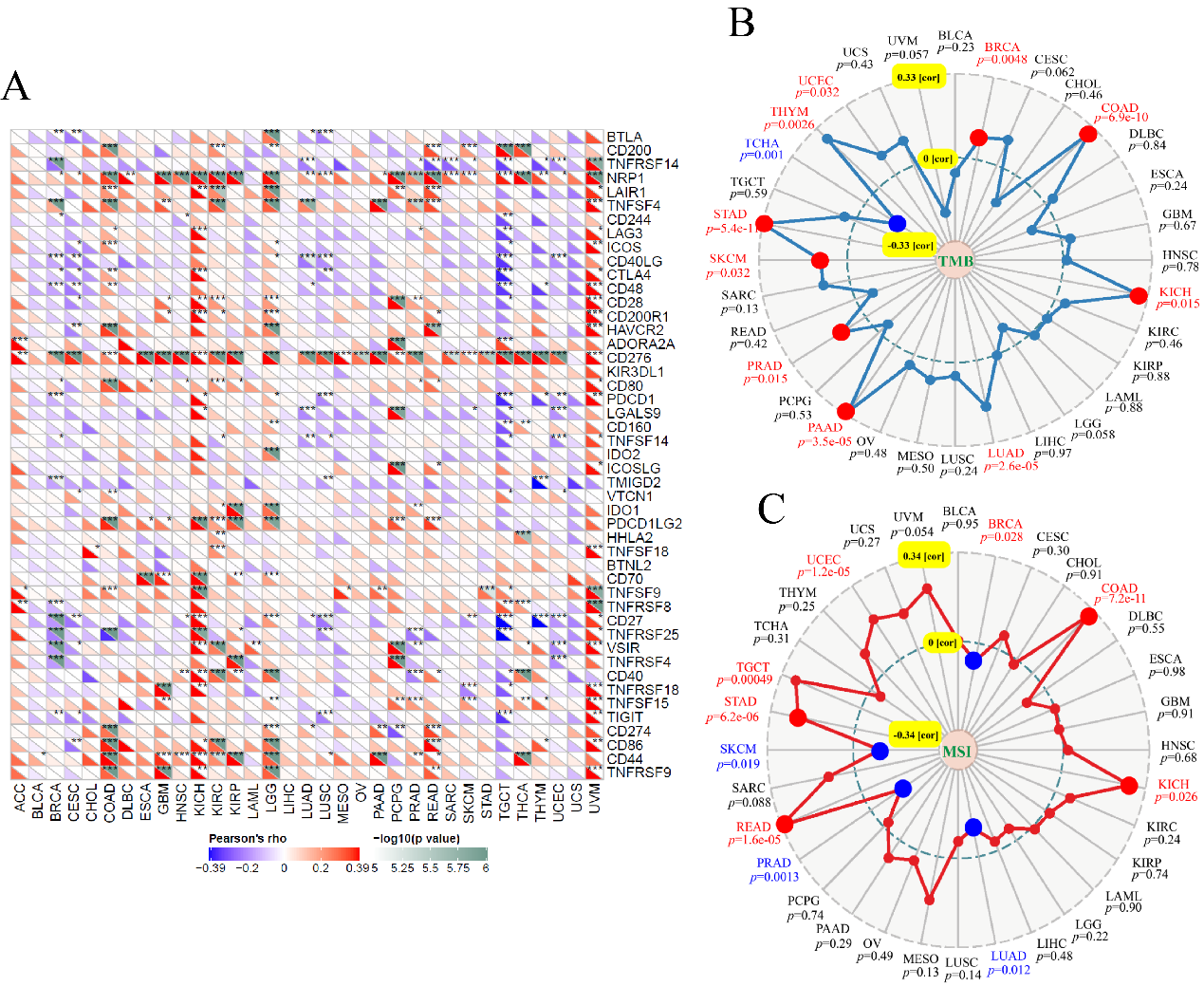
**

**Figure S13**

**
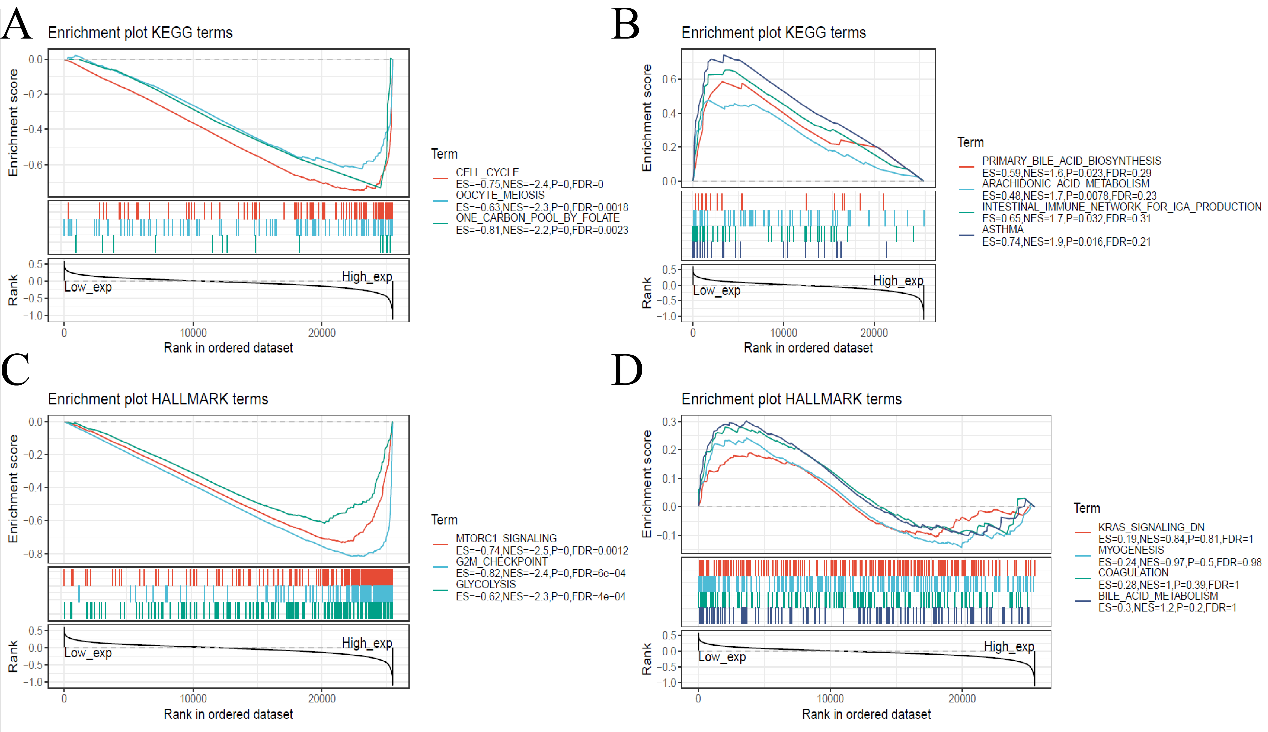
**

**Figure S14**


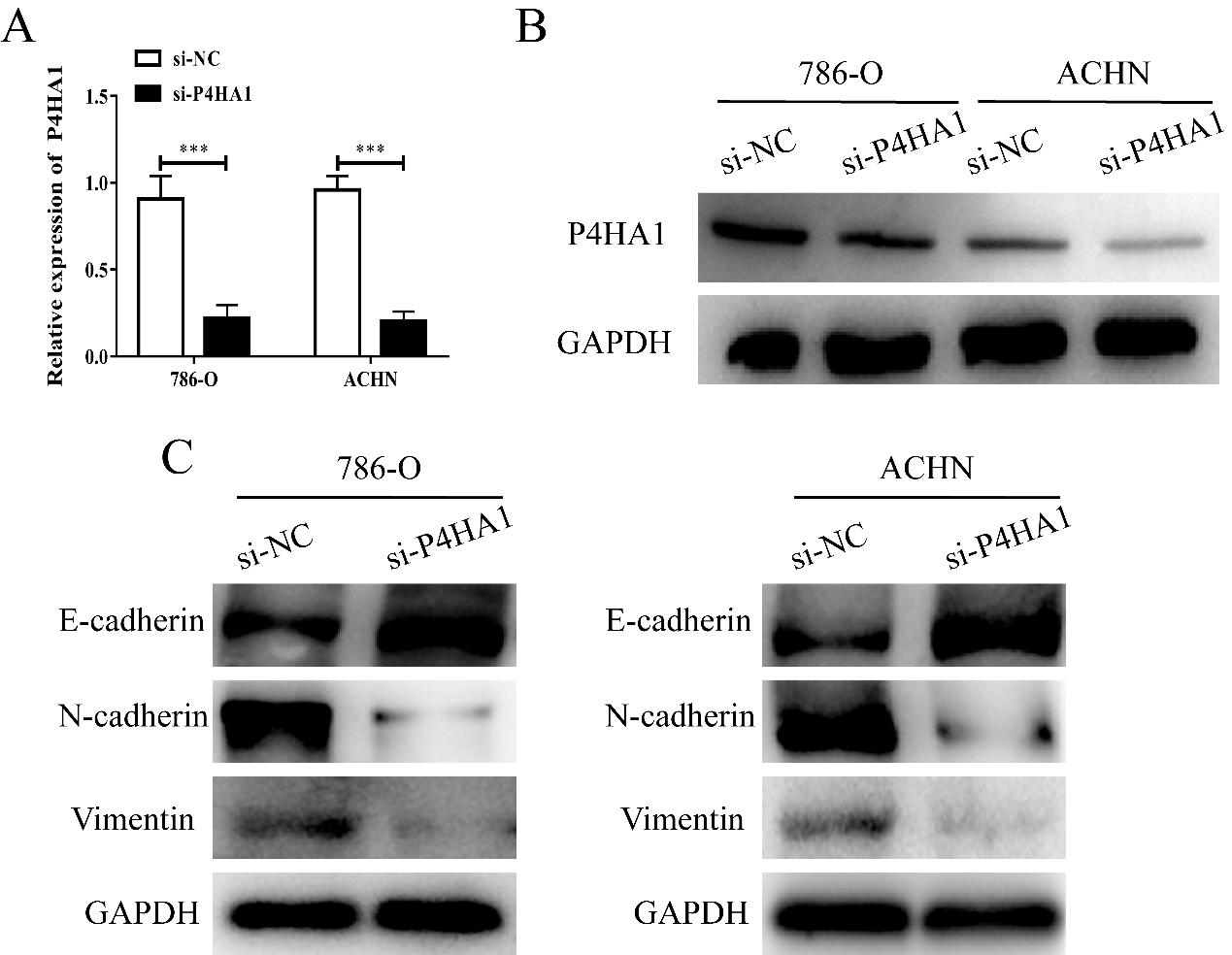


**4. Supplementary tables**

Table S1 Subgroup analysis on the correlation of P4HA1 expression and prognosis of breast cancer cases.

Table S2. Subgroup analysis on the correlation of P4HA1 expression and prognosis of ovarian cancer cases.

Table S3. Subgroup analysis on the correlation of P4HA1 expression and prognosis of lung cancer cases.

Table S4. Subgroup analysis on the correlation of P4HA1 expression and prognosis of gastric cancer cases.

Table S5. Subgroup analysis on the correlation of P4HA1 expression and prognosis of liver cancer cases.

**Reference**

Bonneville, R., Krook, M.A., Kautto, E.A., Miya, J., Wing, M.R., Chen, H.Z., et al. (2017). Landscape of Microsatellite Instability Across 39 Cancer Types. *JCO Precis Oncol* 2017. doi: 10.1200/PO.17.00073.

Kent, W.J., Sugnet, C.W., Furey, T.S., Roskin, K.M., Pringle, T.H., Zahler, A.M., et al. (2002). The human genome browser at UCSC. *Genome Res* 12(6)**,** 996-1006. doi: 10.1101/gr.229102.

Subramanian, A., Tamayo, P., Mootha, V.K., Mukherjee, S., Ebert, B.L., Gillette, M.A., et al. (2005). Gene set enrichment analysis: a knowledge-based approach for interpreting genome-wide expression profiles. *Proc Natl Acad Sci U S A* 102(43)**,** 15545-15550. doi: 10.1073/pnas.0506580102.

| Table S1. Subgroup analysis on the correlation of P4HA1 expression and prognosis of breast cancer cases. | | | | | | |  |  |  |  |
| --- | --- | --- | --- | --- | --- | --- | --- | --- | --- | --- |
|  |  |  |  |  |  |  |  |  |  |  |
| Factor | Subgroup | Sample size | OS | | DMFS | | RFS | | PPS | |
|  |  |  | HR | P | HR | P | HR | P | HR | P |
| **ER status** | ER positive | 3499 | 0.97 | 0.85 | 1.25 | 0.11 | 1.18 | **0.029** | 0.84 | 0.35 |
|  | ER negative | 2168 | 1.49 | **0.019** | 1.38 | **0.015** | 1.43 | **0.00026** | 0.95 | 0.85 |
| **PR status** | PR positive | 1559 | 1.21 | 0.62 | 1.3 | 0.25 | 1.19 | 0.24 | 0.33 | **0.027** |
|  | PR negative | 1989 | 2.18 | **0.0021** | 1.51 | **0.006** | 1.51 | **0.00053** | 0.9 | 0.84 |
| **HER2 status** | HER2 positive | 1273 | 1.21 | 0.31 | 1.12 | 0.51 | 1.48 | **0.00047** | 0.68 | 0.096 |
|  | HER2 negative | 6262 | 1.29 | **0.022** | 1.27 | **0.0065** | 1.35 | **3.20E-07** | 1.02 | 0.88 |
| **Intrinsic subtype** | Basal | 1494 | 2.14 | **0.00012** | 1.82 | **0.00023** | 1.8 | **3.30E-07** | 1.07 | 0.82 |
|  | Luminal A | 3511 | 0.78 | 0.12 | 0.97 | 0.8 | 1.06 | 0.49 | 0.76 | 0.14 |
|  | Luminal B | 2015 | 1.59 | **0.0094** | 1.11 | 0.45 | 1.36 | **0.00064** | 1.15 | 0.5 |
|  | HER2+ | 515 | 0.88 | 0.66 | 0.93 | 0.77 | 1.66 | **0.0051** | 0.71 | 0.37 |
| **Lymph node status** | Lymph node positive | 2153 | 1.3 | 0.11 | 1.39 | **0.011** | 1.38 | **0.00021** | 0.83 | 0.38 |
|  | Lymph node negative | 2829 | 1.11 | 0.53 | 1.37 | **0.013** | 1.26 | **0.0046** | 0.94 | 0.75 |
| **Grade** | Grade 1 | 576 | 0.53 | 0.17 | 1.62 | 0.24 | 0.85 | 0.52 | 0.49 | 0.16 |
|  | Grade 2 | 1795 | 1 | 0.99 | 0.98 | 0.88 | 1.03 | 0.8 | 0.86 | 0.53 |
|  | Grade 3 | 2058 | 1.44 | **0.016** | 1.54 | 0.0013 | 1.48 | **3.80E-05** | 1.14 | 0.48 |
| **TP53 status** | Wild type | 388 | 0.87 | 0.65 | 1.54 | 0.24 | 1.01 | 0.96 | 0.57 | 0.11 |
|  | Mutated | 272 | 1.28 | 0.48 | 1.5 | 0.29 | 1.64 | **0.042** | 0.85 | 0.71 |
| **Pietenpol subtype** | Basal-like 1 | 418 | 2.32 | **0.036** | 1.66 | 0.081 | 1.43 | 0.1 | 0.86 | 0.81 |
|  | Basal-like 2 | 165 | 1.37 | 0.53 | 1.95 | 0.088 | 3.2 | **0.00034** | NA | NA |
|  | immunomodulatory | 462 | 1.92 | 0.11 | 1.34 | 0.3 | 1.36 | 0.18 | 1.21 | 0.72 |
|  | Mesenchymal | 382 | 1.97 | **0.044** | 1.2 | 0.55 | 1.7 | **0.0089** | 0.72 | 0.45 |
|  | Mesenchymal stem-like | 201 | 2.67 | 0.063 | 2.7 | 0.06 | 1.17 | 0.66 | NA | NA |
|  | Luminal androgen receptor | 413 | 0.62 | 0.12 | 1.04 | 0.9 | 1.19 | 0.37 | 0.59 | 0.22 |
|  |  |  |  |  |  |  |  |  |  |  |
| HR, hazard ratio; OS, overall survival; RFS, relapse free survival; DMFS, distant metastasis free survival; ER, Estrogen receptor; PR, Progesterone receptor; HER2, | | | | | | | | | | |
| human epidermal growth factor receptor-2; TP53, Tumor Protein P53; NA, not available data; P value less than 0.05 is shown in bold. | | | | | | | | | | |

| Table S2. Subgroup analysis on the correlation of P4HA1 expression and prognosis of ovarian cancer cases. | | | | | | | |  |
| --- | --- | --- | --- | --- | --- | --- | --- | --- |
|  |  |  |  |  |  |  |  |  |
| Factor | Subgroup | Sample size | OS | | PFS | | PPS | |
|  |  |  | HR | P | HR | P | HR | P |
| **Histology** | Endometrioid | 62 | 1.56 | 0.62 | 0.9 | 0.83 | NA | NA |
|  | Serous | 1232 | 1.12 | 0.16 | 1.19 | **0.018** | 1.12 | 0.19 |
| **Stage** | Stage 1 | 107 | 1.78 | 0.32 | 1.55 | 0.41 | NA | NA |
|  | Stage 2 | 72 | 0.89 | 0.82 | 1.28 | 0.48 | 0.95 | 0.93 |
|  | Stage 3 | 1079 | 1.07 | 0.39 | 1.16 | 0.054 | 1.07 | 0.45 |
|  | Stage 4 | 189 | 1.4 | 0.068 | 1.45 | **0.049** | 1.59 | **0.038** |
| **Grade** | Grade 1 | 56 | 0.5 | 0.15 | 0.92 | 0.87 | NA | NA |
|  | Grade 2 | 325 | 0.99 | 0.94 | 1.13 | 0.39 | 1.02 | 0.9 |
|  | Grade 3 | 1024 | 1.14 | 0.12 | 1.19 | **0.039** | 1.18 | 0.095 |
|  | Grade 4 | 21 | 0.77 | 0.58 | NA | NA | NA | NA |
| **TP53 mutation** | Wild type | 102 | 0.52 | **0.019** | 0.65 | 0.1 | 0.6 | 0.083 |
|  | Mutated | 516 | 1.11 | 0.38 | 1.38 | **0.0041** | 1.15 | 0.27 |
| **Debulk** | Optimal | 802 | 1.06 | 0.6 | 1.14 | 0.19 | 1.06 | 0.65 |
|  | Suboptimal | 536 | 0.97 | 0.77 | 1.19 | 0.1 | 1.04 | 0.75 |
| **Chemotherapy** | Contains platin | 1438 | 1.16 | **0.041** | 1.26 | **0.00046** | 1.14 | 0.13 |
|  | Contains Taxol | 821 | 1.18 | 0.089 | 1.27 | **0.0064** | 1.14 | 0.21 |
|  | Contains Taxol+platin | 804 | 1.16 | 0.14 | 1.25 | **0.011** | 1.12 | 0.29 |
|  | Contains Avastin | 50 | 0.72 | 0.47 | 0.86 | 0.65 | 0.9 | 0.83 |
|  | Contains Docetaxel | 108 | 0.64 | 0.11 | 0.92 | 0.74 | 0.52 | **0.028** |
|  | Contains Gemcitabine | 135 | 0.89 | 0.54 | 0.82 | 0.31 | 0.78 | 0.25 |
|  | Contains Paclitaxel | 248 | 0.79 | 0.3 | 0.91 | 0.59 | 0.86 | 0.57 |
|  | Contains Topotecan | 119 | 0.91 | 0.64 | 0.98 | 0.94 | 0.88 | 0.53 |
|  |  |  |  |  |  |  |  |  |
| HR, hazard ratio; OS, overall survival; PFS, progress free survival; PPS, post progression survival; TP53, Tumor Protein P53; | | | | | | | | |
| NA, not available data; P value less than 0.05 is shown in bold. | | | | | | | | |

| Table S3. Subgroup analysis on the correlation of P4HA1 expression and prognosis of lung cancer cases. | | | | | | |  |  |
| --- | --- | --- | --- | --- | --- | --- | --- | --- |
|  |  |  |  |  |  |  |  |  |
| Factor | Subgroup | Sample size | OS | | FP | | PPS | |
|  |  |  | HR | P | HR | P | HR | P |
| **Histology** | Adenocarcinoma | 865 | 1.07 | 0.59 | 1.22 | 0.21 | 0.89 | 0.63 |
|  | Squamous cell carcinoma | 675 | 0.98 | 0.89 | 1.13 | 0.65 | 0.69 | 0.47 |
| **Stage** | Stage 1 | 652 | 1.22 | 0.14 | 1.05 | 0.81 | 1.01 | 0.97 |
|  | Stage 2 | 320 | 0.86 | 0.43 | 1.22 | 0.46 | 0.55 | 0.071 |
|  | Stage 3 | 70 | 0.96 | 0.88 | NA | NA | NA | NA |
|  | Stage 4 | 4 | NA | NA | NA | NA | NA | NA |
| **Grade** | Grade I | 202 | 1.37 | 0.084 | 1.28 | 0.27 | 1.31 | 0.28 |
|  | Grade II | 310 | 1.37 | **0.048** | 1.43 | 0.089 | 1.18 | 0.52 |
|  | Grade III | 77 | 1.2 | 0.58 | 1.19 | 0.68 | 0.38 | 0.091 |
| **AJCC stage T** | T1 | 475 | 1.58 | **0.0017** | 2.28 | **0.0019** | 0.97 | 0.91 |
|  | T2 | 686 | 1.23 | 0.066 | 1.31 | 0.075 | 1.08 | 0.68 |
|  | T3 | 99 | 0.99 | 0.96 | 1.35 | 0.55 | NA | NA |
|  | T4 | 48 | 1.01 | 0.97 | NA | NA | NA | NA |
| **AJCC stage N** | N0 | 863 | 1.43 | **0.00086** | 1.37 | 0.058 | 0.83 | 0.37 |
|  | N1 | 296 | 1.7 | **0.001** | 1.78 | **0.015** | 1.12 | 0.68 |
|  | N2 | 113 | 0.78 | 0.22 | 1.33 | 0.4 | 0.93 | 0.84 |
| **AJCC stage M** | M0 | 818 | 1.47 | **3.00E-04** | 1.14 | 0.6 | 0.95 | 0.88 |
|  | M1 | 10 | NA | NA | NA | NA | NA | NA |
| **Gender** | Female | 817 | 1.19 | 0.14 | 1.32 | 0.057 | 0.88 | 0.5 |
|  | Male | 1387 | 1.15 | 0.074 | 1.14 | 0.32 | 1.04 | 0.81 |
| **Smoking history** | Exclude those never smoked | 970 | 1.19 | 0.098 | 1.11 | 0.39 | 1.1 | 0.52 |
|  | Only those never smoked | 247 | 1.58 | 0.11 | 1.39 | 0.18 | 0.96 | 0.91 |
| **Surgery success** | Only surgical margins negative | 730 | 1.23 | **0.07** | 1.26 | 0.073 | 1 | 1 |
| **Chemotherapy** | Yes | 178 | 0.86 | 0.48 | 1.39 | 0.12 | 0.89 | 0.62 |
|  | No | 317 | 1.24 | 0.21 | 1.17 | 0.44 | 1.3 | 0.28 |
| **Radiotherapy** | Yes | 73 | 0.85 | 0.56 | 0.93 | 0.78 | 0.8 | 0.47 |
|  | No | 276 | 1.25 | 0.23 | 1.34 | 0.14 | 1.4 | 0.13 |
|  |  |  |  |  |  |  |  |  |
| HR, hazard ratio; AJCC，American Joint Committee on Cancer; OS, overall survival; FP, first progression; | | | | | | | | |
| PPS, post progression survival; NA, not available data; P value less than 0.05 is shown in bold. | | | | | | | | |

| Table S4. Subgroup analysis on the correlation of P4HA1 expression and prognosis of gastric cancer cases. | | | | | | |  |  |
| --- | --- | --- | --- | --- | --- | --- | --- | --- |
|  |  |  |  |  |  |  |  |  |
| Factor | Subgroup | Sample size | OS | | FP | | PPS | |
|  |  |  | HR | P | HR | P | HR | P |
| **Stage** | Stage 1 | 69 | 0.44 | 0.13 | 0.74 | 0.61 | 0 | 0.04 |
|  | Stage 2 | 145 | 0.92 | 0.8 | 0.82 | 0.53 | 0.93 | 0.82 |
|  | Stage 3 | 319 | 0.73 | **0.028** | 0.9 | 0.57 | 0.6 | **0.019** |
|  | Stage 4 | 152 | 0.79 | 0.23 | 0.96 | 0.84 | 0.67 | 0.084 |
| **Stage T** | T1 | 14 | NA | NA | NA | NA | NA | NA |
|  | T2 | 253 | 0.97 | 0.9 | 0.91 | 0.66 | 0.95 | 0.8 |
|  | T3 | 208 | 0.83 | 0.29 | 0.88 | 0.47 | 0.57 | **0.0046** |
|  | T4 | 39 | 0.57 | 0.19 | 0.68 | 0.32 | 0.47 | 0.12 |
| **Stage N** | N0 | 76 | 0.5 | 0.13 | 0.5 | 0.13 | 0.44 | 0.21 |
|  | N1 | 232 | 0.72 | 0.12 | 0.75 | 0.16 | 0.7 | 0.13 |
|  | N2 | 129 | 0.82 | 0.39 | 1.02 | 0.93 | 0.7 | 0.14 |
|  | N3 | 76 | 0.58 | **0.042** | 0.6 | 0.065 | 0.63 | 0.11 |
| **Stage M** | M0 | 459 | 0.83 | 0.19 | 0.88 | 0.33 | 0.63 | **0.0023** |
|  | M1 | 58 | 1.01 | 0.98 | 1.22 | 0.51 | 0.88 | 0.72 |
| **Lauren classification** | Instestinal | 336 | 0.64 | **0.0056** | 0.8 | 0.21 | 0.6 | **0.016** |
|  | Diffuse | 248 | 1.03 | 0.87 | 1.14 | 0.45 | 0.73 | 0.11 |
|  | Mixed | 33 | 0.62 | 0.36 | 1.42 | 0.49 | NA | NA |
| **Differentiation** | Poorly | 166 | 0.88 | 0.52 | 1.17 | 0.49 | 0.85 | 0.62 |
|  | Moderately | 67 | 1.45 | 0.26 | 1.59 | 0.14 | 1.42 | 0.44 |
|  | Well | 32 | 0.86 | 0.72 | NA | NA | NA | NA |
| **Gender** | Female | 244 | 0.82 | 0.26 | 0.89 | 0.55 | 0.6 | **0.017** |
|  | Male | 566 | 0.67 | **0.00022** | 0.71 | **0.0056** | 0.61 | **0.00023** |
| **Perforation** | Yes | 4 | NA | NA | NA | NA | NA | NA |
|  | No | 169 | 1.19 | 0.4 | 1.24 | 0.27 | 1.02 | 0.95 |
| **Treatment** | Surgery alone | 393 | 1.09 | 0.55 | 1.13 | 0.38 | 0.72 | **0.038** |
|  | 5-Fu based adjuvant | 157 | 1.33 | 0.1 | 1.38 | 0.063 | 1.2 | 0.31 |
|  | other adjuvant | 80 | 1.05 | 0.92 | 0.91 | 0.8 | 0.86 | 0.74 |
| **HER2** | positive | 424 | 0.91 | 0.47 | 0.91 | 0.56 | 0.67 | **0.023** |
|  | negative | 641 | 0.66 | **0.00035** | 0.76 | **0.039** | 0.61 | **0.00083** |
|  |  |  |  |  |  |  |  |  |
| HR, hazard ratio; OS, overall survival; FP, first progression; PPS, post progression survival; | | | | | | | | |
| HER2, human epidermal growth factor receptor-2; NA, not available data; P value less than 0.05 is shown in bold. | | | | | | | | |

| Table S5. Subgroup analysis on the correlation of P4HA1 expression and prognosis of liver cancer cases. | | | | | | |  |  |  |  |
| --- | --- | --- | --- | --- | --- | --- | --- | --- | --- | --- |
|  |  |  |  |  |  |  |  |  |  |  |
| Factor | Subgroup | Sample size | OS | | PFS | | RFS | | DDS | |
|  |  |  | HR | P | HR | P | HR | P | HR | P |
| **Stage** | Stage 1 | 171 | 0.94 | 0.85 | 0.79 | 0.36 | 1 | 0.99 | 0.75 | 0.21 |
|  | Stage 2 | 86 | 0.63 | 0.25 | 0.85 | 0.58 | 0.93 | 0.83 | 0.62 | 0.39 |
|  | Stage 3 | 85 | 1.08 | 0.81 | 0.63 | 0.095 | 0.66 | 0.17 | 0.72 | 0.36 |
|  | Stage 4 | 4 | NA | NA | NA | NA | NA | NA | NA | NA |
| **Grade** | Grade 1 | 55 | 0.73 | 0.6 | 1.51 | 0.3 | 1.64 | 0.33 | 0.73 | 0.6 |
|  | Grade 2 | 177 | 0.78 | 0.34 | 0.72 | 0.13 | 0.75 | 0.24 | 0.49 | **0.041** |
|  | Grade 3 | 122 | 0.85 | 0.59 | 0.68 | 0.13 | 0.8 | 0.41 | 1 | 1 |
|  | Grade 4 | 12 | NA | NA | NA | NA | NA | NA | NA | NA |
| **AJCC_T** | T1 | 181 | 0.91 | 0.74 | 0.77 | 0.3 | 0.94 | 0.8 | 0.79 | 0.57 |
|  | T2 | 94 | 0.67 | 0.29 | 0.77 | 0.36 | 0.82 | 0.53 | 0.71 | 0.48 |
|  | T3 | 80 | 0.8 | 0.46 | 0.69 | 0.19 | 0.73 | 0.31 | 0.71 | 0.35 |
|  | T4 | 13 | NA | NA | NA | NA | NA | NA | NA | NA |
| **Vascular invasion** | None | 205 | 0.83 | 0.47 | 0.95 | 0.82 | 0.93 | 0.77 | 0.9 | 0.77 |
|  | Micro | 93 | 0.76 | 0.49 | 0.49 | **0.015** | 0.64 | 0.16 | 0.64 | 0.42 |
|  | Macro | 16 | NA | NA | NA | NA | NA | NA | NA | NA |
| **Gender** | Male | 250 | 0.84 | 0.44 | 0.78 | 0.17 | 0.79 | 0.24 | 0.74 | 0.3 |
|  | Female | 121 | 1.48 | 0.17 | 1.43 | 0.17 | 1.36 | 0.3 | 1.73 | 0.13 |
| **Race** | White | 184 | 0.95 | 0.84 | 0.86 | 0.46 | 1.1 | 0.67 | 0.9 | 0.72 |
|  | Bkack | 17 | NA | NA | NA | NA | NA | NA | NA | NA |
|  | Asian | 158 | 0.7 | 0.24 | 0.7 | 0.13 | 0.65 | 0.097 | 0.7 | 0.37 |
| **Sorafenib treatment** | Treated | 30 | 0.81 | 0.7 | 0.92 | 0.83 | 1.43 | 0.43 | 0.76 | 0.61 |
| **Alcohol consumption** | Yes | 117 | 0.89 | 0.73 | 0.72 | 0.2 | 0.66 | 0.16 | 0.84 | 0.64 |
|  | none | 205 | 1.07 | 0.77 | 0.79 | 0.24 | 0.94 | 0.78 | 0.88 | 0.69 |
| **Hepatitis virus** | Yes | 153 | 0.88 | 0.7 | 0.81 | 0.37 | 1.17 | 0.54 | 0.88 | 0.75 |
|  | none | 169 | 1.05 | 0.85 | 0.78 | 0.26 | 0.79 | 0.36 | 0.98 | 0.94 |
|  |  |  |  |  |  |  |  |  |  |  |
| HR, hazard ratio; AJCC，American Joint Committee on Cancer; OS, overall survival; PFS, progress free survival; | | | | | | | | | | |
| RFS, relapse free survival; DSS, disease specific surviva; NA, not available data; P value less than 0.05 is shown in bold. | | | | | | | | | | |
